# Observation of Bose-Einstein condensates of excitons in a bulk semiconductor

**DOI:** 10.1038/s41467-022-33103-4

**Published:** 2022-09-14

**Authors:** Yusuke Morita, Kosuke Yoshioka, Makoto Kuwata-Gonokami

**Affiliations:** 1 Department of Physics, Graduate School of Science, The University of Tokyo, 7-3-1 Hongo, Bunkyo-ku, Tokyo, 113-0033 Japan; 2 Photon Science Center, Graduate School of Engineering, The University of Tokyo, 2-11-16 Yayoi, Bunkyo-ku, Tokyo, 113-8656 Japan

**Keywords:** Bose-Einstein condensates, Semiconductors, Exotic atoms and molecules, Optical physics

## Abstract

An unambiguous observation of the Bose-Einstein condensation (BEC) of excitons in a photoexcited bulk semiconductor and elucidation of its inherent nature have been longstanding problems in condensed matter physics. Here, we observe the quantum phase transition and a Bose-Einstein condensate appearing in a trapped gas of 1*s* paraexcitons in bulk Cu_2_O below 400 mK, by directly visualizing the exciton cloud in real space using mid-infrared induced absorption imaging that we realized in a dilution refrigerator. Our study shows that the paraexciton condensate is undetectable by conventional luminescence spectroscopy. We find an unconventionally small condensate fraction of 0.016 with the spatial profile of the condensate well described by mean-field theory. Our discovery of this new type of BEC in the purely matter-like exciton system interacting with a cold phonon bath could pave the way for the classification of its long-range order, and for essential understanding of quantum statistical mechanics of non-equilibrium open systems.

## Abstract (web summary)

Bose-Einstein condensate of excitons is expected in photo-excited bulk semiconductors, but a direct experimental evidence has been lacking. Here the authors report the observation of a condensate of 1s paraexcitons in Cu_2_O using real-space mid-infrared absorption imaging realized in a dilution refrigerator.

## Introduction

Bose–Einstein condensation (BEC) is a quantum statistical phase transition in the matter, manifesting itself as a macroscopic occupation in the ground state. Experimental and theoretical investigations on BEC in dilute atomic gases have substantially promoted the understanding of the nature of condensates since the realization of atomic BEC^1–3^.

The BEC of exotic atoms is one of the major challenges in modern physics that has yet to be achieved^4,5^. An exciton is a type of exotic composite particle similar to positronium^6,7^ and muonium. It is formed in photoexcited semiconductors or insulators as a bound electron–hole pair with a finite lifetime. Excitons that are decoupled from the radiation field are purely matter-like quasi-particles with a corpuscular nature and therefore the only counterpart of exotic atoms in solids. The ability to generate a high-density gas of excitons by laser excitation and subsequently cool the gas via exciton–phonon interactions with a cold phonon bath has been widely considered beneficial to realizing exotic BEC.

The BEC phase is one of a rich variety of matter phases that an electron–hole system forms depending on experimentally controllable parameters such as gas temperature and density. Other phases, including an electron–hole plasma^8^ and electron–hole droplets^9^, have been observed. However, the direct observation of a matter-like exciton condensate in a bulk semiconductor has been a highly sought-after “holy grail” in low-temperature physics since its initial proposal in 1962^10^. The observation of an exciton condensate also directly leads to solving a variety of inherent problems in solid state physics: quantum statistical phenomena originating from finite fermionic components in the commutation relation^11^ and the quantum statistical mechanics of a non-equilibrium open system in thermal contact with phonons.

Indirect excitons in coupled quantum well structures are important two-dimensional exotic atoms in artificially grown solids. Spatially resolved photoluminescence measurements from indirect excitons revealed the appearance of a macroscopically ordered state^12–15^ in the ground state. They addressed the formation of quasi-long-range order corresponding to the Berezinskii–Kosterlitz–Thouless transition in the two-dimensional system. On the other hand, condensation of microcavity exciton polaritons^16,17^ which is strongly coupled to the radiation field also has been studied. Though, it requires the realization of exciton BEC, which is decoupled from the radiation field in a bulk semiconductor, to fully address the fundamental question raised by Kohn and Sherrington^18^ regarding whether the exciton condensate exhibits an off-diagonal long-range order^19^. The macroscopic occupation of such excitons in the ground state of an engineered trap potential whose wave function is well characterized in both momentum space and real space has not been reported.

The 1*s* paraexcitons in cuprous oxide (Cu_2_O) are one of the most promising candidates^20,21^ for exciton BEC in a bulk semiconductor because of their long lifetime^20,22^. The hydrogen-like 1*s* exciton state is split into a triply degenerate orthoexciton state and a lower-lying singly degenerate paraexciton state, depending on the spin configuration of an electron and a hole. The 1*s* paraexciton is the pure spin triplet state, which results in very weak coupling with the radiation field. 1*s* paraexcitons are experimentally well known to have an extremely long lifetime of over several hundred nanoseconds. This long lifetime is an important prerequisite for the preparation of cold paraexcitons through thermal contact with the lattice of Cu_2_O.

Studies of paraexciton BEC at liquid helium temperatures around 2 K began in the 1990s^23,24^. The density and temperature of 1*s* excitons were conventionally estimated by luminescence spectroscopy^21,23,24^. However, the two-body loss coefficients of 1*s* orthoexcitons^25^ and 1*s* paraexcitons^22^ are so large that the density of both species was far below that estimated by lineshape analyses. The “quantum saturation”^26,27^ and fulfilment of the BEC criteria^24^ were later proven to originate from integrated spectra of spatially inhomogeneous dilute classical gases with various local densities and temperatures^25^. The target temperature for paraexciton BEC is sub-1 K with reduced BEC transition densities to avoid two-body inelastic collisions^22^. Short-lived 1*s* orthoexcitons cannot reach such a low temperature. A method for effective collection of 1*s* paraexcitons in a strain-induced trap potential^28,29^ was developed for the realization of exciton BEC because the paraexciton diffusion at the temperature of paraexcitons sub-1 K hinders the efficient accumulation of paraexcitons. Employment of the method and cooling of trapped paraexcitons below 1 K led to the observation of a phenomenon suggesting the BEC transition of paraexcitons and the formation of the condensate with large instability induced by two-body inelastic collisions^30^. This phenomenon is called a “relaxation explosion”^31^, which was originally found in BEC of gas of spin-polarized atomic hydrogen in a magnetic trap^32^. The formation of a stable condensate requires further reduction of two-body inelastic collisions that induce relaxation explosion. Previously, this was achieved via the application of a dilution refrigerator^33–35^ that enabled realization of trapped paraexcitons with a reduced BEC transition density of 10^15^ cm^−3^ at a temperature of sub-100 mK^33^. Interactions between trapped paraexcitons and transverse acoustic phonons under the strain field, which functioned as the trap potential, critically contributed to the reduction of the exciton temperature well below 1 K.

From the history of the search for exciton BEC, a common question arises: what makes direct detection of the condensate so difficult? Various answers have been suggested, including a possible instability caused by the efficient formation of biexcitons at the corresponding BEC transition densities^36^ although the existence of biexcitons in Cu_2_O has not been confirmed. Other studies suggested restrictions in the detection of a stable exciton condensate by luminescence spectroscopy^37^, which has been a conventional method aiming for the detection of exciton BEC^23,24^.

**Fig. 1:**
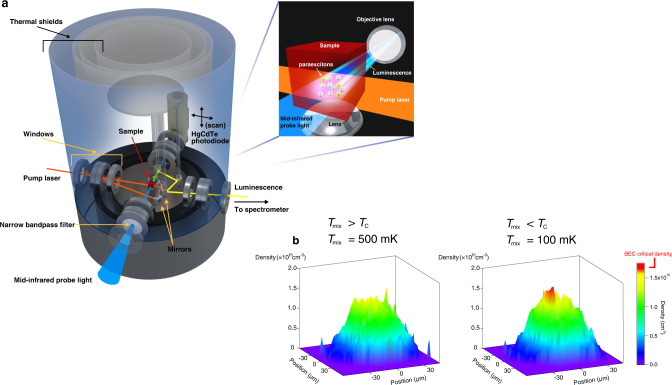
A novel setup for absorption imaging and appearance of the condensate. **a** Experimental setup inside the cryogen-free dilution refrigerator, showing the optical paths of the pump laser (orange solid line), luminescence (yellow solid line), mid-infra-red probe light (blue solid line), and location of the sample material (red cube). The optical components (mirrors and mirror holders), thermal shields (blue and grey cylinders), windows, narrow bandpass filters, and a nitrogen-cooled HgCdTe photodiode (beige cylinder) are also labelled. We measured the spatially resolved differential transmission that was the absorption image using the photodiode set on a translation-motorized stage for scanning. The spatial resolution of the imaging system was 7.8 μm (FWHM). (upper right panel) Schematic illustration of the physical processes involved for paraexcitons in the sample: excitation, luminescence, and absorption. The excitation beam (orange solid line) propagated in the sample (red cube). A paraexciton (yellow sphere) consists of one electron (blue sphere) and one hole (red sphere). We detected paraexcitons by either luminescence (yellow shade) or the differential transmission of the probe light (blue shade). An objective lens set behind the sample collected luminescence from paraexcitons. The probe beam also propagated through the objective lens. We applied inhomogeneous stress using a lens set under the sample. **b** Emergence of the exciton condensate in the density distribution at *P*_pump_ = 1.6 mW when *T*_mix_ < 400 mK. Left (Right) panel shows the spatial distribution of the paraexciton density measured by induced absorption imaging at *T*_mix_ = 500 mK (100 mK). The vertical axis shows the paraexciton density, and the horizontal plane shows the position in the trap potential. The density of a local dense signal at *T*_mix_ = 100 mK exceeds the BEC transition density of 1.7 × 10^15^ cm^−3^.

Here, we perform mid-infra-red induced absorption imaging associated with the 1*s*–2*p* transition of paraexcitons for the quantitative detection of exciton BEC at dilution temperatures. Absorption measurements have attracted attention as a sensitive detection method well suited for dark paraexcitons in Cu_2_O because the 1*s*–2*p* transition is dipole allowed. They allow us to determine the absolute density of paraexcitons, which is an essential parameter for the evaluation of quantum statistical mechanics, using the transition dipole moment^22,38^. The absorption imaging^37^, as is routinely done in cold-atom experiments, enables us to extract the spatial density distribution of trapped paraexcitons and to observe the formation of the paraexciton condensate in situ with the appearance of the local dense region in the spatial density distribution. However, the 1*s*–2*p* resonance wavelength of 9.8 μm lies in the centre of the spectrum of the blackbody radiation at room temperature^39^, so that incoming thermal flow to the coldest part in a dilution refrigerator after passing through windows (shown in Fig. 1a), maintaining at dilution temperatures is difficult. Our key technical achievement (see the “Methods” section) is the successful suppression of incoming thermal radiation in the dilution refrigerator by the use of narrow bandpass filters as windows and properly restricting their aperture sizes, which enabled us to conduct mid-infra-red-induced absorption at dilution temperatures down to 64 mK.

We report the observation of the paraexciton condensate in a trapping potential at dilution temperatures below 400 mK using absorption imaging. Figure 1b shows one of the decisive pieces of evidence for the formation of paraexciton BEC. The figure shows the spatial distributions of the density of trapped 1*s* paraexcitons for 1.6 mW excitation power when the temperature of the mixing chamber (*T*_mix_) is 500 and 100 mK. A localized dense signal appears around the centre of the cloud at *T*_mix_ = 100 mK where the condensate fraction is 1.2%. In addition, during this imaging process, we simultaneously performed conventional luminescence spectroscopy to complement the paraexciton density findings and prove that the paraexciton condensate is undetectable by luminescence spectroscopy.

## Results and discussion

### Luminescence spectroscopy

**Fig. 2:**
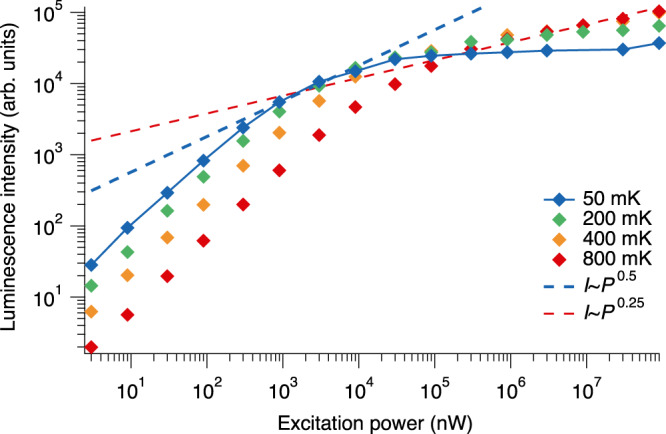
Luminescence intensity of trapped 1*s* paraexcitons as a function of excitation power at varying temperatures of the mixing chamber. The dashed blue (red) line shows the excitation power dependence proportional to the 0.5th (0.25th) power of the temperature.

In order to measure the luminescence intensity of paraexcitons at the bottom of the strain-induced trap potential, we first observed a typical spatially resolved luminescence spectrum via the direct recombination of 1*s* paraexcitons, which is slightly allowed by the strain field, as shown in Supplementary Fig. 1. Note that transmission of the mid-infra-red probe light was prohibited during this experiment. Figure 2 shows the signal intensity that increases in a region of interest (see the “Methods” section) as a function of excitation power (*P*_pump_) when *T*_mix_ is 50, 200, 400, and 800 mK. The excitation power ranged from 30 nW to 90 mW.

It also shows that the luminescence intensity does not increase linearly with the excitation power typically above 90 μW. The luminescence intensity (*I*) as a function of excitation power at high paraexciton densities indicates the specific square-root power-law dependence (*I* *∝* *P*_pump_^0.5^), which originates from the two-body collision-induced loss (two-body loss coefficient^22^: about 10^−16^ cm^3^/ns). For example, the blue dashed line in Fig. 2 shows that the excitation power dependence of the luminescence intensity changes from a linear relation to the specific dependence at 900 nW < *P*_pump_ < 9 μW when *T*_mix_ = 50 mK. However, the intensity at *P*_pump_ ≥ 300 μW hardly increases for *T*_mix_ ≤ 200 mK. This intensity saturation has never been observed. The red dashed line shows the strongest saturation previously reported^28^ (*I* *∝* *P*_pump_^0.25^), where dense paraexcitons are generated 400 μm away from the bottom of a trap, whereas we generated only <10 μm away from the bottom of the trap.

The strong saturation of the luminescence suggests the transition to a quantum degenerate regime of paraexcitons at sub-Kelvin temperatures. In the one-photon direct emission process, the momentum conservation requires that the translational momentum of paraexcitons is the same as the momentum of the emitted photon (*p*_0_ = 3 × 10^−27^ kg m/s) (see Supplementary Note 4). In a quantum statistical distribution at temperatures below 400 mK, paraexcitons mainly relax to smaller momentum states than *p*_0_ by bosonic-stimulated scattering when the total number of paraexcitons increases. This increase in the number of paraexcitons in low-momentum states brings about a decrease in the net emission efficiency, resulting in saturation of the luminescence intensity. Moreover, the decrease in the temperature causes a reduction in the required density for the formation of quantum statistical distributions, which results in saturation at weaker excitation powers. Indeed, Fig. 2 shows that saturation of the luminescence intensity starts at weaker excitation powers as the temperature decreases. Our numerical simulation reproduces the saturation as well as the approximate excitation power where the saturation begins, i.e., *P*_pump_ = 9 μW (see Supplementary Note 4). To obtain the absolute density of paraexcitons at strong excitation powers, we performed mid-infra-red-induced absorption measurements.

### Measurement of induced absorption

**Fig. 3:**
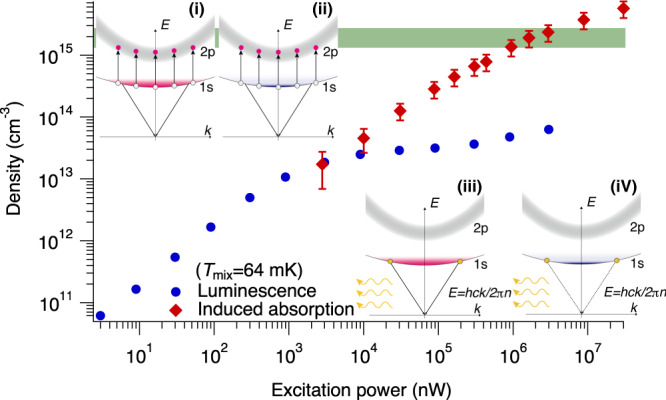
Density of trapped 1*s* paraexcitons as a function of excitation power. Red diamonds show absolute densities of 1*s* paraexcitons measured by induced absorption at various excitation powers when *T*_mix_ is 64 mK. Blue circles show densities of 1*s* paraexcitons estimated solely from the luminescence intensity at various excitation powers. The red bars show the accuracy of measuring the density of 1*s* paraexcitons (see Supplementary Note 5). The green shaded bar shows the BEC transition density of 1.7 ± 0.6 × 10^15^ cm^−3^ at the measured paraexciton temperature of 170 ± 30 mK, where the paraexciton mass is 2.61 ± 0.04*m*_0_ (ref. 40) (2.4 ± 0.3*m*_0_ (ref. 41)). **i**, **ii** Schematic illustration of the induced absorption process associated with the transition of paraexcitons from the 1*s* state (white circles) to the 2*p* state (red circles). The transition of 1*s* paraexcitons with any energy and momentum is resonant with probe light because the linewidth of the 2*p* state (grey region) is large at 1.2 meV. **iii**, **iv** Schematic illustration of the direct recombination process of 1*s* paraexcitons (yellow circles) with emission of photons (yellow arrows). **i**, **iii** The statistical distribution of 1*s* paraexcitons consists of non-quantum degenerate states at a low density. **ii**, **iv** The statistical distribution of 1*s* paraexcitons contains quantum degenerate states at a high density.

We performed 1*s*–2*p*-induced absorption measurements of paraexcitons at *P*_pump_ ≥ 3 μW and *T*_mix_ = 64 mK. This allowed us to determine the absolute density of paraexcitons at the bottom of the trap potential using the transition dipole moment^37^. The luminescence intensity was recorded simultaneously with these induced absorption measurements to both confirm the density values and determine whether the paraexciton condensate is detectable by luminescence spectroscopy. The 1*s* paraexciton temperature (*T*_ex_) measured by luminescence spectroscopy at *T*_mix_ = 64 mK was 170 ± 30 mK during this experiment. The red diamonds in Fig. 3 are the absolute densities determined from the induced absorption amounts at various excitation powers.

Meanwhile, the luminescence intensity is proportional to the paraexciton density when it is relatively low, below 1% of the BEC transition density. Therefore, we can relate the luminescence intensity to the estimated paraexciton density at *P*_pump_ = 90 nW (see Supplementary Note 5). The blue dots in Fig. 3 show that the density estimated from the luminescence intensity agrees with the absolute density determined from the induced absorption measurement at *P*_pump_ = 3 μW. However, the luminescence intensity saturates at *P*_pump_ > 30 μW (see Supplementary Note 5) while the absolute density continues to increase. Moreover, they show that the paraexciton density at excitation powers above 1.6 mW exceeds the 1.7 ± 0.6 × 10^15^ cm^−3^ BEC transition density, where the paraexciton mass is 2.61 ± 0.04*m*_0_ (see ref. 40) (2.4 ± 0.3*m*_0_ (see ref. 41), *m*_0_ is the free electron mass). The strong saturation supports our view that quantum degenerate paraexcitons below 400 mK, including Bose–Einstein condensates, cannot luminesce.

### Absorption imaging

**Fig. 4:**
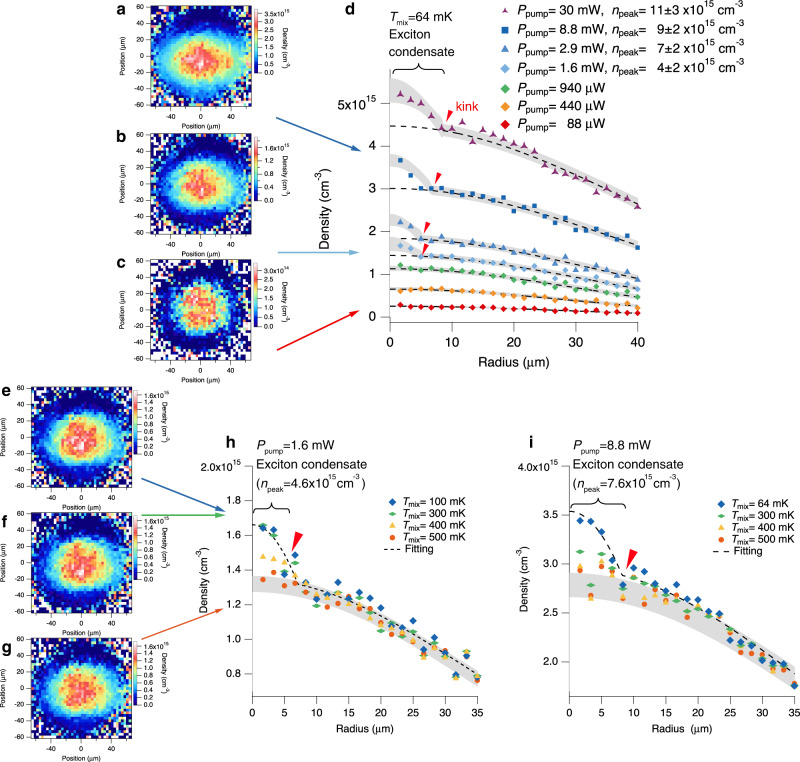
Paraexciton density measured by induced absorption imaging. **a–c** Spatial distributions of the paraexciton density for different excitation powers at *T*_mix_ = 64 mK: **a** *P*_pump_ = 8.8 mW, **b** *P*_pump_ = 1.6 mW, and **c** *P*_pump_ = 88 μW. **d** Radial profiles of the averaged density (see Supplementary Note 5) at various excitation powers for *T*_mix_ = 64 mK. The black dashed curves show the Gaussian distribution fit to the tail of the radial profiles. The grey shaded curves show the bimodal distribution fit to the radial profile of the density. The term *n*_peak_ denotes the estimated peak density of trapped paraexcitons. **e–g** Spatial distribution of the paraexciton density measured by induced absorption imaging at *P*_pump_ = 1.6 mW for different temperatures of the mixing chamber: **e** *T*_mix_ = 100 mK, **f** *T*_mix_ = 300 mK, and **g** *T*_mix_ = 500 mK. **h**, **i** Radial profiles of the averaged density at various temperatures of the mixing chamber when **h** *P*_pump_ = 1.6 mW and **i** *P*_pump_ = 8.8 mW. The grey shaded curves show the prediction bands estimated by fitting the radial profile at *T*_mix_ = 500 mK with a Gaussian distribution. The black dashed curves show the bimodal distribution fit to the radial profile of the density at **h** *T*_mix_ = 100 mK and **i** *T*_mix_ = 64 mK. The term *n*_peak_ denotes the estimated peak density of trapped paraexcitons.

We performed mid-infra-red-induced absorption imaging associated with the 1*s*–2*p* transition of paraexcitons. Figure 4a–c shows the spatial distribution of the 1*s* paraexcitons density along the [100] and [011] crystal axes at *P*_pump_ = 88 μW, 1.6, and 8.8 mW when *T*_ex_ = 170 ± 30 mK. These figures show that a localized dense signal appears around the centre of the cloud at *P*_pump_ = 1.6 and 8.8 mW. The characterization of this signal strongly suggests the appearance of a Bose–Einstein condensate of excitons. The absolute density of this localized dense signal in Fig. 4b is 1.9 × 10^15^ cm^−3^, which is above the 1.7 × 10^15^ cm^−3^ BEC critical density at 170 mK. Moreover, the full width at half maximum (FWHM) of the signal is 8.0 ± 1.4 μm (7.8 ± 1.3 μm) along the [011] ([100]) crystal axis at *P*_pump_ = 1.6 mW. These widths are smaller than that of the thermal gas (FWHM) of 39 μm at 170 mK in the trap potential. Ignoring the two-body interaction, the theoretical density width of the ground state in the trap potential determined by the trap frequency along the [011] ([100]) crystal axis is 1.5 μm (1.3 μm), while the lateral resolution is 7.8 μm. Therefore, we conclude that the localized signal is a resolution-limited absorption image of a Bose–Einstein condensate. Accurate estimations of the exciton density and the exciton temperature (see Supplementary Note 2) enabled us to quantitatively identify the signal. Note that we tentatively set the positional origin for the data analysis at the top of the positional distribution of the thermal component shown in Fig. 4c. The position of the localized dense signal in the definition of position is −10 μm. Our preliminary studies suggest that the defined origin does not necessarily coincide with the minimum position of the potential because of the position-dependent shift of the 1*s*–2*p* transition energy in the inhomogeneous strain distribution. Therefore, the position of the signal could be the bottom of the trap potential.

Considering the trap potential anisotropy, we obtained the radial profile of the averaged density by angle-averaging the data (see Supplementary Note 5) at various excitation powers. The dashed curves in Fig. 4d show the Gaussian distribution fit to the tail of the radial profiles. The radial profile at *P*_pump_ ≥ 1.6 mW distinctly exhibits a non-Gaussian distribution, which confirms that *P*_pump_ = 1.6 mW is the threshold for the appearance of the localized dense signal around the bottom of the trap potential. In Fig. 4d the density at the bottom of the trap potential is 1.2 × 10^15^ cm^−3^ at *P*_pump_ = 940 μW which is just below the threshold. This density is within the precision error for the BEC critical density of 1.7 ± 0.6 × 10^15^ cm^−3^. These facts strongly suggest the observation of the BEC condensate.

To check whether this threshold temperature agrees with the critical temperature for BEC, we measured the paraexciton density for the localized dense signal by fitting the radial profile with a bimodal distribution^42^ . (For more information on the validity of bimodal fitting, see Supplementary Note 5.) Fig. 4h (Fig. 4i) shows that this bimodal distribution fits the lowest temperature, *T*_mix_ = 100 mK (64 mK), radial profile well (black dashed curve). The estimated peak densities of 4.6 ± 0.7 × 10^15^ cm^−3^ (7.6 ± 1.8 × 10^15^ cm^−3^) at *P*_pump_ = 1.6 mW (8.8 mW) correspond to the critical density for BEC at 330 ± 80 mK (460 ± 100 mK) (see Supplementary Note 5 for peak calculations). Therefore, the threshold-like temperature dependence of the locally condensed signal strongly indicates that the emergence of the localized dense signal corresponds to the formation of the paraexciton condensate.

We observed the exciton condensate as a localized dense signal in the trap potential, as also observed in atomic BEC. In the following, we discuss the many similarities and differences between paraexciton BEC and atomic BEC.

First, we observed the excitation power dependence of the width of the paraexciton condensate. The local dense signal, which corresponds to the paraexciton condensate, appears at the centre of the cloud in Fig. 4b, c. As previously described, at *P*_pump_ = 1.6 mW the signal has a resolution-limited width (FWHM) of 8.0 ± 1.4 μm (7.8 ± 1.3 μm) along the [011] ([100]) crystal axis. Meanwhile, at *P*_pump_ = 8.8 mW the width (FWHM) of the local dense signal becomes asymmetric, 19 ± 7 μm (13 ± 3 μm) along the [011] ([100]) crystal axis and the estimated peak density of the signal is 7.6 ± 1.8 × 10^15^ cm^−3^. Because repulsive interactions between Bose particles increase the size of the condensate in a trap, the excitation power dependence suggests that the exciton–exciton interaction is repulsive. Here, we used the Gross–Pitaevskii equation to estimate the scattering length of paraexcitons to be 4 ± 2 nm, which accounts for the observed asymmetric spatial widths along the [011] and [100] crystal axes at *P*_pump_ = 8.8 mW (see Supplementary Note 6). Previous works based on quantum Monte Carlo simulations reported paraexciton scattering lengths of 2.1*a*_B_ ~ 1.4 nm^43^ and 3*a*_B_ ~ 2 nm^44^ (*a*_B_ is the Bohr radius of paraexcitons). The ~1 nm discrepancy between our estimated scattering length and previously reported scattering lengths requires further investigation. Nevertheless, the non-trivial agreement of the order of magnitude between them implies that the Gross–Pitaevskii equation is applicable to describing exciton BEC.

**Fig. 5:**
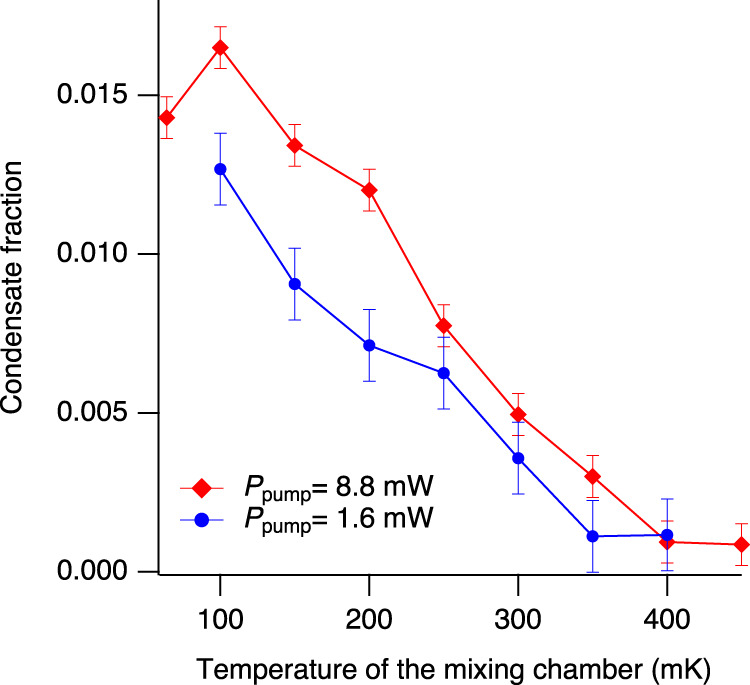
Condensate fraction as a function of the temperature of the mixing chamber. Blue and red circles represent the condensate fraction for 1.6 and 8.8 mW excitation powers, respectively. Red and Blue bars represent experimental uncertainty for each point determined by the dynamic range of the absorption measurements.

Second, as shown in Fig. 5, we evaluated the temperature dependence of the condensate fraction at *P*_pump_ = 1.6 and 8.8 mW from the absolute numbers of paraexcitons in the exciton condensate and the thermal gas. The condensate fraction has a non-zero value below the estimated critical temperature of 330 ± 80 mK (460 ± 100 mK) for BEC at *P*_pump_ = 1.6 mW (8.8 mW) and increases in a nearly linear fashion with decreasing temperature of the mixing chamber. The maximum condensate fraction is 0.016 (0.013) at *P*_pump_ = 8.8 mW (1.6 mW). The number of paraexcitons in the condensate is 2 × 10^6^ (6 × 10^6^) at *P*_pump_ = 1.6 mW (8.8 mW). Meanwhile, the number of paraexcitons in the thermal component is 1.7 × 10^8^ (4.8 × 10^8^) at all temperatures when *P*_pump_ = 1.6 mW (8.8 mW). Therefore, as *P*_pump_ is increased from 1.6 to 8.8 mW, the condensate fraction hardly increases from 0.013 to 0.016 while the total number of paraexcitons increases to 2.5 times its original magnitude. We note that the temperature *T*_ex_ of the quantum degenerate exciton gases cannot be measured by luminescence spectroscopy because, as explained above, a major portion of the gas does not luminesce. Therefore, we plotted the condensate fraction as a function of the temperature of the mixing chamber *T*_mix_, although some discrepancy between *T*_mix_ and *T*_ex_ may arise.

For comparison, we note that the condensate fraction of ideal Bose particles^42^ and atomic gases with repulsive interactions^45^ converge to 1 at absolute zero. Therefore, the maximum condensate fraction of paraexcitons is two orders of magnitude smaller than that expected for an ideal Bose gas. Qualitatively similar phenomena are known for the case of atomic gases with attractive interactions^46,47^ or spin-polarized atomic hydrogen^32^, where the maximum condensate fraction is similarly much smaller than unity because the condensate is unstable.

The expansion of the paraexciton condensate suggests that paraexcitons have repulsive interactions. However, the maximum condensate fraction of paraexcitons is well below that of the atomic condensate with repulsive interactions. This difference may be interpreted as a manifestation of the composite boson in a non-equilibrium open system with a balance between generation and decay, such as the finite lifetime of paraexcitons, two-body inelastic collisions between paraexcitons, and suppression of the thermal relaxation processes. For instance, the formation and stability of condensate in such a composite system have been studied theoretically in refs. 27,48. Especially, theoretical calculations^48^ suggest that a large condensate fraction is prohibited in a system where there is a balance between the generation and decay of particles.

In conclusion, we observed a Bose–Einstein condensate of 1*s* paraexcitons in a bulk crystal of semiconductor Cu_2_O at *T*_mix_ < 400 mK. The achievement of absorption imaging in the mid-infra-red region at dilution temperatures of 64 mK enabled observation of the paraexciton condensate as a locally condensed signal in a trap potential. Our study revealed that the paraexciton condensate is undetectable by conventional luminescence spectroscopy. Application of the mean-field approximation to the paraexciton condensate allowed us to find that the exciton–exciton interaction is repulsive and that the scattering length is 4 ± 2 nm. Unconventionally, the maximum condensate fraction that we observed is 0.016, which is two orders of magnitude smaller than that expected for an ideal Bose gas. The discovery of this new type of BEC opens a brand-new research field of quantum statistical mechanics of a non-equilibrium open system coupled with a thermal bath.

The classification of the long-range order that the exciton condensate gains in bulk semiconductors has been a controversial mystery in the field of solid-state physics^18,19^. The development of time-resolved absorption imaging based on our setup may reveal the dynamics of coherence acquisition and condensate formation, as well as the transport properties. For example, it will allow us to revisit the unresolved physical origin of the ballistic exciton transport observed in photovoltaic measuements^49,50^. Future works on the paraexciton condensate shall address the fundamental question of whether quantum statistical phenomena such as the BEC to Bardeen–Cooper–Schrieffer (BCS) crossover^51–53^ occur in the non-equilibrium open system. Simultaneous observation of mid-infra-red-induced absorption and the far-infra-red dielectric response upon high-density excitation will be helpful for investigating such quantum many-body phases in ultracold electron–hole systems in a semiconductor.

## Methods

We describe our sample, excitation light, probe light for absorption imaging, dilution refrigerator, and designs for luminescence spectroscopy and induced absorption imaging in the following text.

### Sample

We used a naturally grown pure single crystal of Cu_2_O mined in Namibia. The sample dimensions were 5.3 × 5.3 × 8.0 mm^3^. The surface of our sample consisted of the [100], [011], and $[01\bar{1}]$ crystal planes. The lowest exciton state in Cu_2_O, i.e., the so-called “yellow series exciton state”, is a well-known typical Wannier–Mott exciton state with a large binding energy of 150 meV (ref. 54). The lowest exciton state consists of an electron in the lowest conduction band (Γ^+^_6_) and a hole in the highest valence band (Γ^+^_7_). It shows a hydrogen-like Rydberg series. The lowest exciton state, which has the principal quantum number *n* = 1, is split into two states: triply degenerate orthoexciton states (Γ^+^_5_) and a nondegenerate paraexciton state (Γ^+^_2_). The energy of the paraexciton state is 12 meV lower than that of the orthoexciton state. This 1*s* paraexciton state is the ground state of the exciton in Cu_2_O. Under no external field, the parity and spin selection rules forbid optical direct recombination of paraexcitons for all orders of transitions. Meanwhile, the phonon-assisted recombination processes of paraexcitons are weakly allowed. Application of stress to the crystal makes the direct recombination process weakly allowed because the finite strain field couples paraexcitons with an exciton state in the green series. The weakness of the coupling between paraexcitons and the radiation field brings about a long paraexciton lifetime. We determined the lifetime to be 600 ± 20 ns using time-resolved luminescence spectroscopy^55^, as explained in the following. We note that an exceptionally long lifetime (13 μs (ref. 20)) was reported in Cu_2_O. The 2*p* paraexciton state is 128 meV higher than the 1*s* paraexciton state in a strain-free crystal. The 1*s*–2*p* transition of paraexcitons is dipole allowed.

We applied inhomogeneous stress via the Hertzian contact^28,56,57^ between the [100] crystal surface and the spherical surface of a lens (BK7, radius of curvature = 15.57 mm). The inhomogeneous stress results in an inhomogeneous strain field that acts as a trap potential for 1*s* paraexcitons. The trap potential is nearly harmonic in three dimensions. We evaluated the trap frequencies to be 8.4, 8.4, and 12.4 MHz along the [011], $[01\bar{1}]$, and [100] crystal axes, respectively. The strain field shifts the energy of 1*s* paraexcitons, resulting in a shift of the peak photon energy of the luminescence from 1*s* paraexcitons via the direct recombination process^58^. We estimated the magnitude of the strain field at the bottom of the trap potential from the shift in the peak energy of the luminescence. Moreover, we calculated the spatial distribution of the inhomogeneous strain field and the resulting energy shift to evaluate the trap frequencies shown above. We confirmed the calculated potential curve by comparing it with spatially resolved luminescence spectra.

### Excitation light to generate high-density paraexcitons

We generated a sufficient number of 1*s* paraexcitons for the realization of paraexciton BEC. However, the direct creation of 1*s* paraexcitons is inefficient because the coupling between paraexcitons and the radiation field is very weak. Therefore, we generated orthoexcitons via the Γ^−^_3_-longitudinal-optical (LO)-phonon-assisted absorption process, which is dipole allowed. Paraexcitons are generated via the orthoexciton–paraexciton conversion process. The conversion time depends on the lattice temperature and strain field. Conversion times of 7–12 ns (6 ns) have been reported under the same applied stress as in the present experiment at dilution temperatures^55^ (at a 2 K lattice temperature^59^). These values are at least one order of magnitude smaller than the lifetime of 1*s* paraexcitons, resulting in sufficient accumulation of paraexcitons within the lifetime.

We generated 1*s* orthoexcitons using the CW fibre Raman laser (Optoquest Co., Ltd. FPS-606 1 W) as excitation light. The laser wavelength was 606.1 nm for selective generation of 1*s* orthoexcitons near the bottom of the trap potential. The maximum output of the laser was 1 W. The CW fibre Raman laser exhibits excellent long-term stability of the output power compared with a dye laser, which has been conventionally used as excitation light for the generation of 1*s* orthoexcitons via the Γ^−^_3_-LO-phonon-assisted absorption process. The high stability of the CW fibre Raman laser enabled us to perform absorption imaging associated with the 1*s*–2*p* transition of paraexcitons for a long period of time, resulting in absorption images with a good noise-to-signal ratio.

We used the first-order diffraction of an acousto-optic modulator (AOM) with a high resonance frequency (200 MHz, IntraAction Corp., ATM-200C1) to chop the CW beam. The excitation beam was introduced to the sample along the [011] crystal axis. A focusing lens (focal length = 300 mm) set outside the refrigerator focused the excitation beam on the trap bottom. The spot size (FWHM) of the focused excitation light was 30 μm. We controlled the peak intensity of the chopped beam using neutral density filters.

### Probe light for absorption imaging

We employed the CW quantum cascade laser (Daylight Solutions Inc., TLS-001-PL-9.5 μm) as a probe light resonant with the 1*s*–2*p* transition of paraexcitons for the induced absorption measurements. The laser is tunable within a range of 9.05–11.43 μm. In our absorption imaging experiments, we chose a wavelength of 9.82 μm, which is around the peak energy of the induced absorption spectrum, as explained in Supplementary Note 1. The wavelength was measured by a wavelength meter (Bristol Instruments, 621 series). The typical output power was 100 mW. We used an AOM (1207B-3, ISOMET Co.) to chop the CW probe light, as was done for the excitation light. We reduced the peak power to below 10 mW using a neutral density filter. We confirmed that the heat induced by the probe light hardly increased the paraexciton temperature within an error range of 30 mK, as explained in Supplementary Note 2. A focusing lens (focal length = 300 mm) set outside the refrigerator loosely focused the probe beam on the exciton cloud. A focusing lens (focal length = 300 mm) set outside the refrigerator focused the probe light on the sample. The entire trapped paraexciton cloud, whose typical width (FWHM) was 39 μm, was illuminated by the probe light whose beam width (FWHM) was 400 μm at the sample.

### Dilution refrigerator and designs for luminescence spectroscopy and induced absorption imaging

We constructed our apparatus based on a cryogen-free dilution refrigerator (Oxford Instruments, DR-400) for mid-infra-red-induced absorption imaging and luminescence spectroscopy at dilution temperatures. Our apparatus has the following four features.

1. The refrigerator has a high cooling power of 400 μW at a temperature of 100 mK. Our refrigerator unavoidably allows a finite amount of thermal radiation transmission into the sample stage despite the careful design for optical access, as explained below. The high cooling power compensates for this incoming heat, leading to the realization of a base temperature of 64 mK.
2. The cryogen-free system allows running for a long period of time. The long-term operation enabled us to perform absorption imaging with a good noise-to-signal ratio. One image typically took several hours to obtain depending on the 1*s* paraexciton density.
3. We set a ZnSe meniscus lens (focal length: 5 mm, effective aperture: 6 mm) as an objective lens close to the sample to measure the intensity of the mid-infra-red probe light and to observe the luminescence from the trapped 1*s* paraexcitons, as explained below. A large numerical aperture (NA: 0.45) is an important prerequisite for absorption imaging with a high spatial resolution of 7.8 μm (FWHM). We finely adjusted the position of the lens at low temperatures using a piezoelectric motor attached to the sample stage.
4. The special design for the refrigerator minimizes the mechanical vibration and the position drift of the sample stage, which was experimentally confirmed to be sufficiently small (much less than our spatial resolution of 7.8 μm). This facilitated our high-resolution imaging.

A schematic of the experimental setup for induced absorption imaging and luminescence spectroscopy for 1*s* paraexcitons is shown in Fig. 1a. The Cu_2_O crystal (red cube in Fig. 1a) was placed on a sample stage at the centre of the dilution refrigerator. We attached windows to the shields of the refrigerator that allowed optical access to the sample stage in four directions. The windows in two directions allowed transmission of the excitation light (orange solid line in Fig. 1a) and luminescence from paraexcitons (yellow solid line in Fig. 1a) in the visible region. The excitation beam propagated in the crystal along the sample [011] axis. The excitation light wavelength was 606.1 nm and the luminescence wavelength was 614.1 nm. The windows in the other two directions allowed transmission of the probe light (blue solid line in Fig. 1a) for induced absorption imaging. However, because the 9.8 μm probe light wavelength is at the peak of the blackbody radiation at room temperature^36^ the windows also transmit thermal radiation to the sample stage. To reduce incoming thermal radiation, we carefully designed the windows by minimizing the numerical aperture and using a narrow bandpass filter (FWHM ~ 100 nm, manufactured by Northumbria Optical Coatings Ltd.) as the window material. The limited area of the windows was similar to that of the probe beam at the window (~5 × 5 mm^2^). This specialized design for the windows and the high cooling power of the cryogen-free dilution refrigerator facilitated the realization of a 64 mK minimum base temperature. We further controlled the base temperature in the range of 64–800 mK with a heater attached to the mixing chamber plate. We controlled the lattice temperature via control of the base temperature. Note that the paraexciton temperature is slightly higher than the lattice temperature^30^ because paraexcitons have imperfect thermal contact with the lattice.

### Details of our measurements

We describe our measurements of the spatially resolved luminescence spectrum via the direct recombination of 1*s* paraexcitons and mid-infra-red-induced absorption associated with the 1*s*–2*p* transition of paraexcitons in the following text.

### Luminescence spectroscopy

We detected the spatially resolved luminescence spectrum of 1*s* paraexcitons via the direct recombination process. We set the ZnSe meniscus lens (focal length: 5 mm, effective aperture: 6 mm) as an objective lens close to the sample. It allowed us to collect luminescence from paraexcitons with a large solid angle. The luminescence images were magnified 15 times. We employed the 50-cm imaging spectrometer to resolve the photon energy. When we set the entrance slit of the spectrometer to 30 μm, the energy resolution of the spectrometer was 50 μeV, which was the best energy resolution. We spatially resolved the luminescence along the [100] crystal axis. We detected the time-integrated luminescence spectrum using an electron multiplying charge-coupled device (CCD) camera (Andor Technology, DU970N-BV) and detected the time-resolved luminescence spectrum for measurement of the paraexciton lifetime using an intensified CCD camera (ICCD, iStar DH334T, Andor Technology).

We used the spatially resolved luminescence spectrum to plot Figs. 2 and 3. We fixed the slit width to 30 μm to obtain the best energy resolution. A typical spatially resolved luminescence spectrum of 1*s* paraexcitons is shown in Supplementary Fig. 1. The signal at approximately 2018.2 meV is the luminescence from the direct recombination process of 1*s* paraexcitons. We processed the time-integrated luminescence intensity by integrating the signal intensity spectrally over 60 μeV and spatially over 16 μm. This integration was done around the pixel with the peak signal intensity to evaluate the contribution from the paraexcitons at the bottom of the trap potential. We set the exposure time to several tens of minutes depending on the signal strength. Figure 2 shows the signal intensity in the region of interest as a function of the excitation power at *T*_mix_ = 50–800 mK.

### 1*s*–2*p*-induced absorption measurements

We chopped the excitation light and the probe light using AOMs in our measurements of the differential transmission. The timing of turning the AOMs on and off was controlled by electrical pulses from function generators (AFG3102, Tektronix Inc.). The repetition frequency of the AOM for the excitation light (probe light) was 500 Hz (1 kHz). The pulse width was 5 μs (2 μs). The time delay for the rising edge of the probe with respect to that of the excitation was 2 μs.

The absorption image obtained was the spatially resolved differential transmission induced by absorption of the trapped paraexcitons. We used the ZnSe meniscus lens as the objective lens for mid-infra-red absorption imaging. The absorption image was magnified 15 times, and the spatial resolution of the imaging system was 7.8 μm (FWHM). We measured the differential transmission using a nitrogen-cooled HgCdTe (MCT) detector (Kolmar Technologies Inc., KLD-0.1-J1/11/DC, active area of 0.1 × 0.1 mm^2^). We set the MCT detector on the translation-motorized stage for scanning. When we performed the absorption imaging, we scanned the detector along the [100] and [011] crystal axes at an interval of 50 μm.

To detect dilute paraexcitons at a density as low as 10^13^ cm^−3^ or below, we realized a wide dynamic range better than 3 × 10^−4^ by limiting the detection bandwidth to 300 kHz and using a synchronized 16-bit AD converter. Typically, 3 h was needed to take an absorption image.

## Supplementary information

Supplementary information

## Data Availability

The data used in this study have been deposited in the figshare with the identifier 10.6084/m9.figshare.20526900.
