# Supplementary material for: Observation of Bose-Einstein condensates of excitons in a bulk semiconductor
Source: Nat Commun. 2022 Sep 14;13:5388. doi: 10.1038/s41467-022-33103-4 (PMC9474864; doi:10.1038/s41467-022-33103-4)
Supplement: Supplementary file 1 — Supplementary information [file 41467_2022_33103_MOESM1_ESM.pdf]

Supplementary information of

"Observation of Bose-Einstein condensates of excitons in a bulk semiconductor"

Yusuke Morita[1], Kosuke Yoshioka[1,2*], Makoto Kuwata-Gonokami[1*]

[1]Department of Physics, Graduate School of Science, The University of Tokyo, 7-3-1 Hongo, Bunkyo-ku, Tokyo 113-0033, Japan.

[2]Photon Science Center, Graduate School of Engineering, The University of Tokyo, 2-11-16 Yayoi, Bunkyo-ku, Tokyo 113-8656, Japan.

*Corresponding author. Email: gonokami@phys.s.u-tokyo.ac.jp, yoshioka@fs.t.u-tokyo.ac.jp

Supplementary Figures

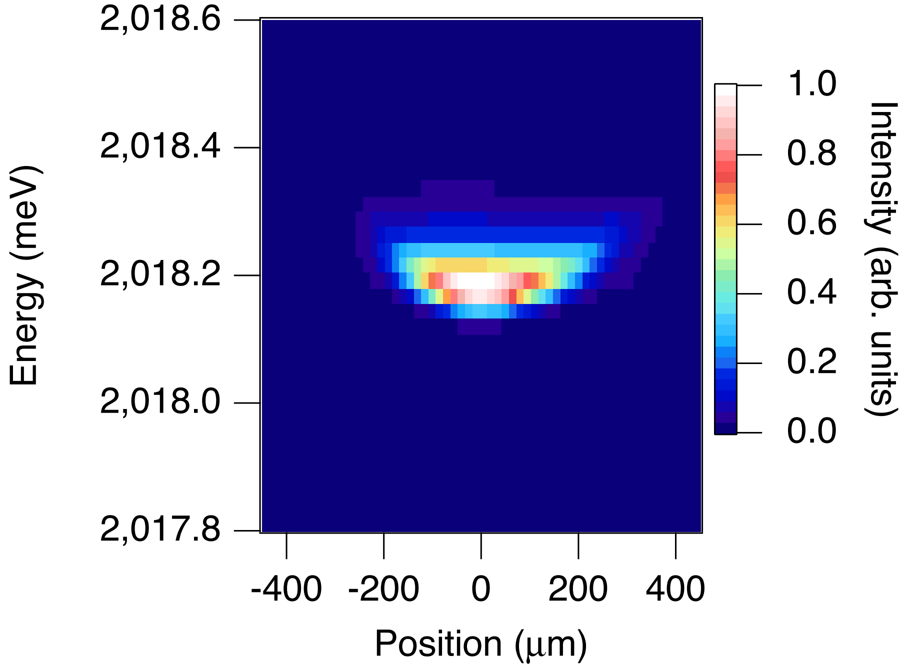

Supplementary Figure 1 | Typical spatially resolved luminescence spectrum emitted from trapped paraexcitons via the direct recombination process at T_{mix}= 55 mK. The excitation power, exposure time, accumulation number and energy resolution were 30 μW, 3 s, 100 and 50 μeV, respectively.

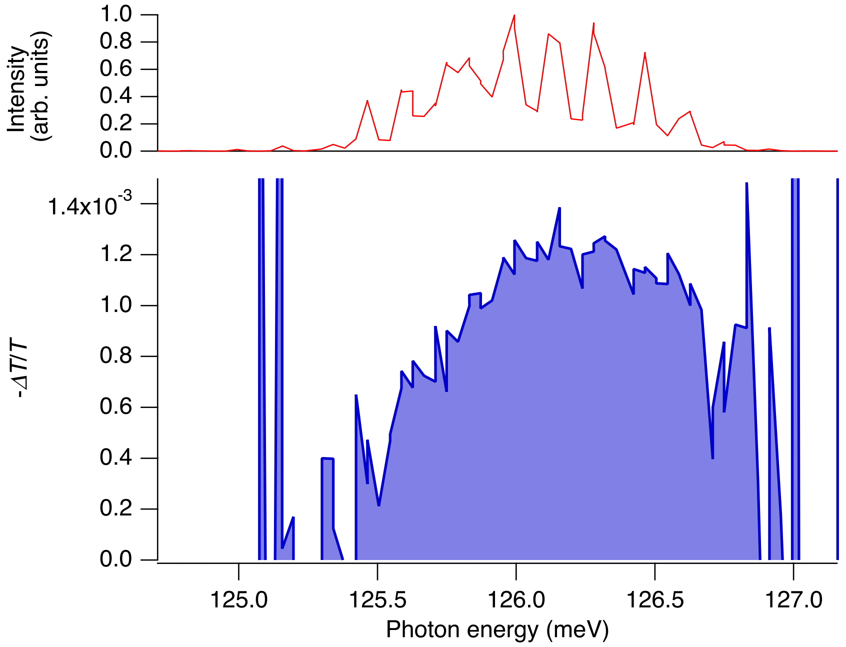

Supplementary Figure 2 | 1s-2p induced absorption spectrum of 1s paraexcitons at the bottom of the trap potential (blue curve) and corresponding intensity of the transmitted probe light (red curve) at T_{mix}= 64 mK and P_{pump}=300 μW.

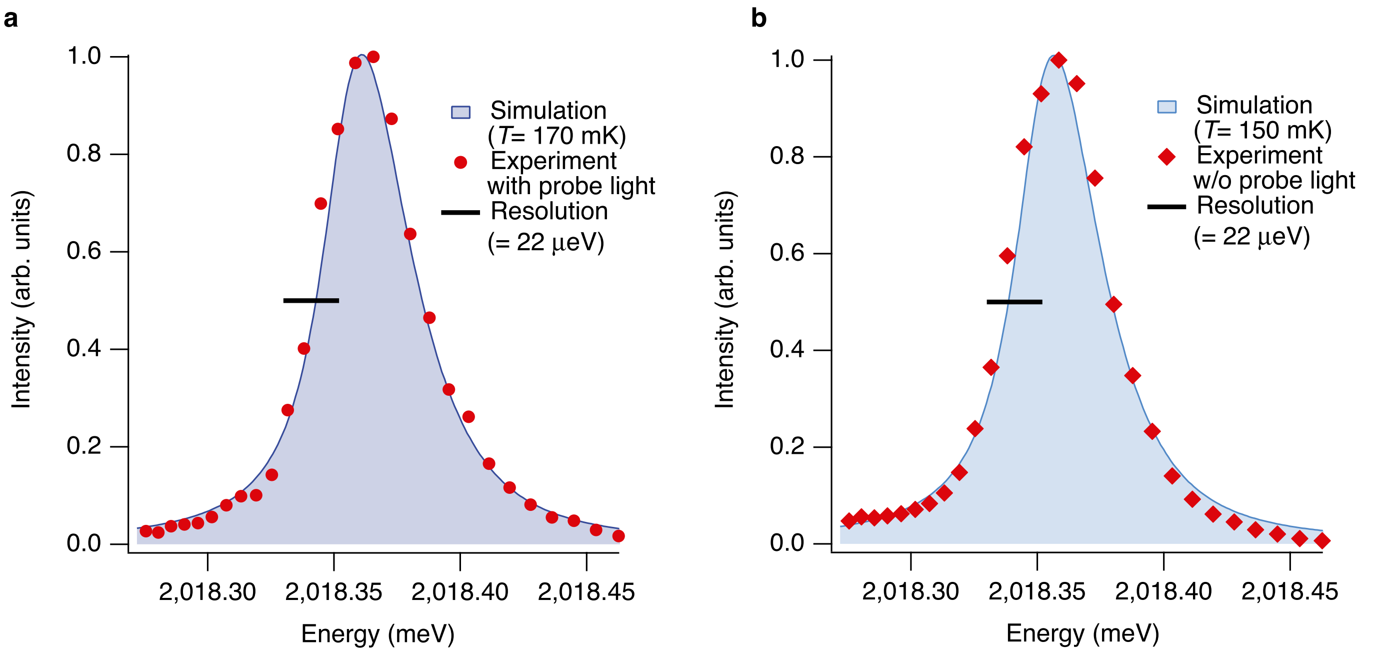

Supplementary Figure 3 | High-resolution luminescence spectra of 1s paraexcitons at P_{pump}=90 nW and T_{mix}= 64 mK.

(a) Red circles show the experimental luminescence spectrum with the probe light. The purple shaded area shows the calculated luminescence spectrum at 170 mK that best reproduces the experimental data.

(b) Red diamonds show the experimental luminescence spectrum without the probe light. The blue shaded area shows the calculated luminescence spectrum at 150 mK that best reproduces the experimental data.

The black bar shows the energy resolution of our measurements of the luminescence spectra.

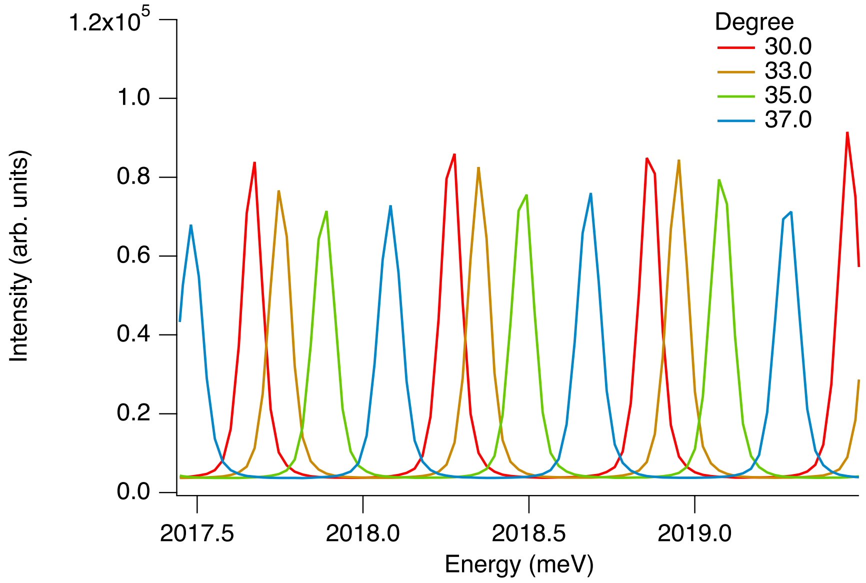

Supplementary Figure 4 | Typical spectra of a white light which passes through the etalon.

The red, yellow, green, and blue curves show the spectrum at the angle of 30 degrees, 33 degrees, 35 degrees and 37 degrees, respectively.

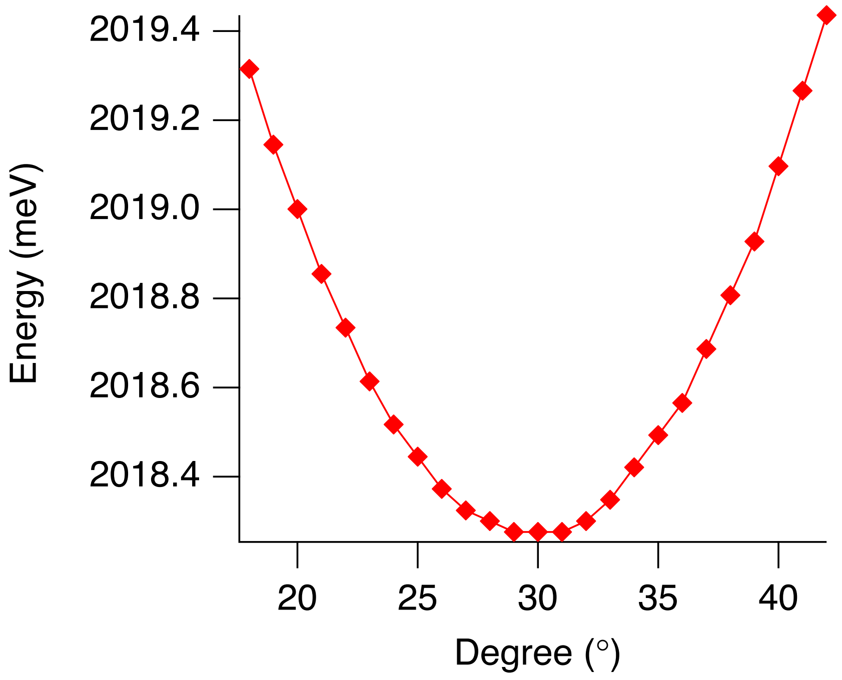

Supplementary Figure 5 | Photon energy of the transmitted luminescence as a function of the angle of the solid etalon.

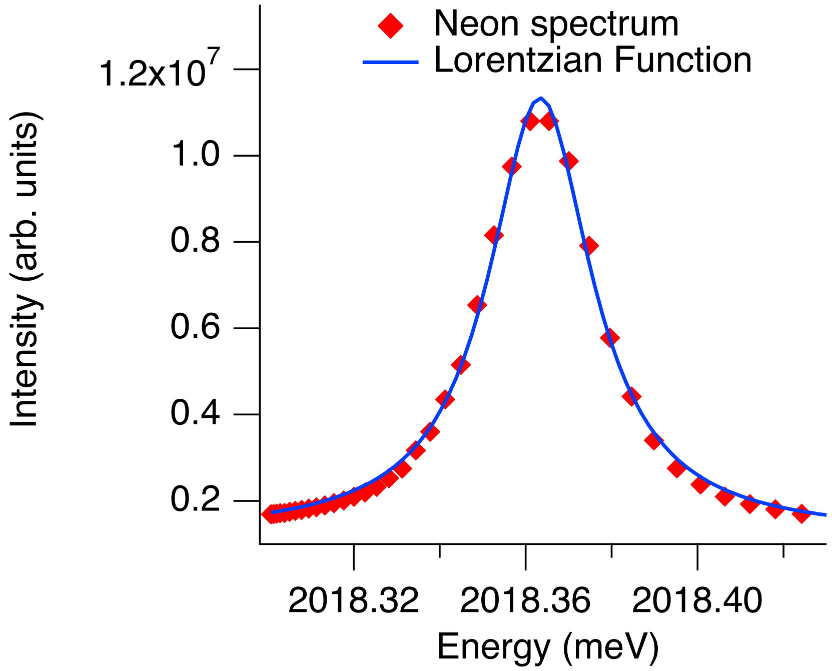

Supplementary Figure 6 | High-resolution spectrum of neon lamp. The red diamonds show the experimental spectrum of a neon lamp. The blue curve shows the Lorenz function that reproduces the experimental spectrum.

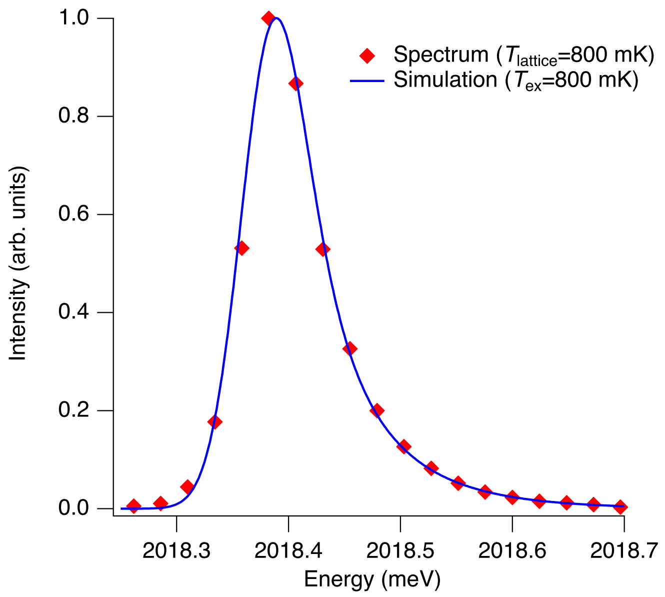

Supplementary Figure 7 | Luminescence spectrum emitted from trapped paraexcitons via the direct recombination process at $T_{lattice}$= 800 mK. The red diamonds show the experimental luminescence spectrum. The blue curve shows the calculated luminescence spectrum ($I_{conv}(E, T$=800 mK)) that best reproduces the experimental data.

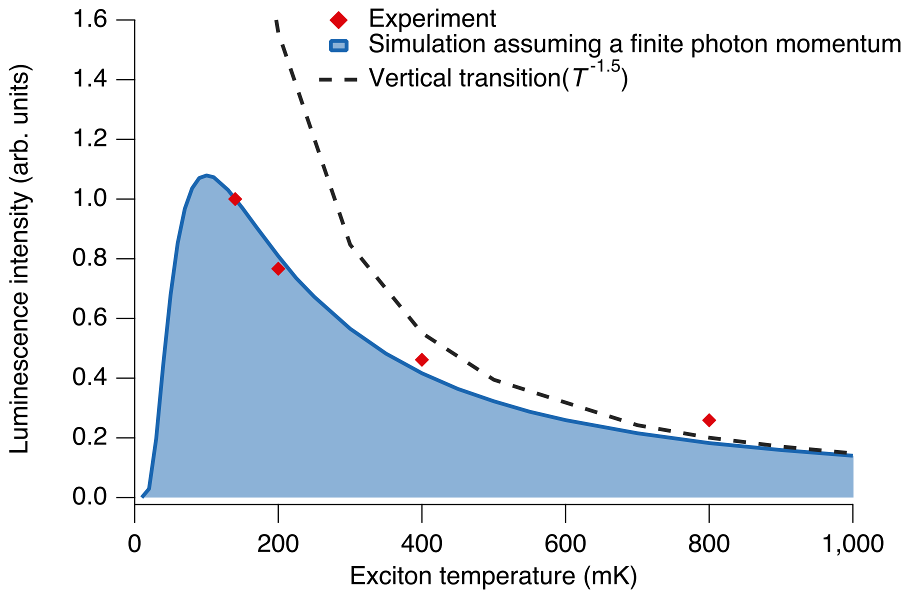

Supplementary Figure 8 | Luminescence intensity as a function of paraexciton temperature at an excitation power of 90 nW. The red diamonds show the experimental results. The black dashed curve shows the calculated intensity based on the assumption of the vertical transition. The curve shows that the temperature dependence is proportional to the -1.5th power of the temperature (~ $T^{-1.5}$). Our calculation (blue curve) assumes that only the paraexcitons that have a translational momentum as large as the finite momentum of the emitted photon can participate in the direct recombination process.

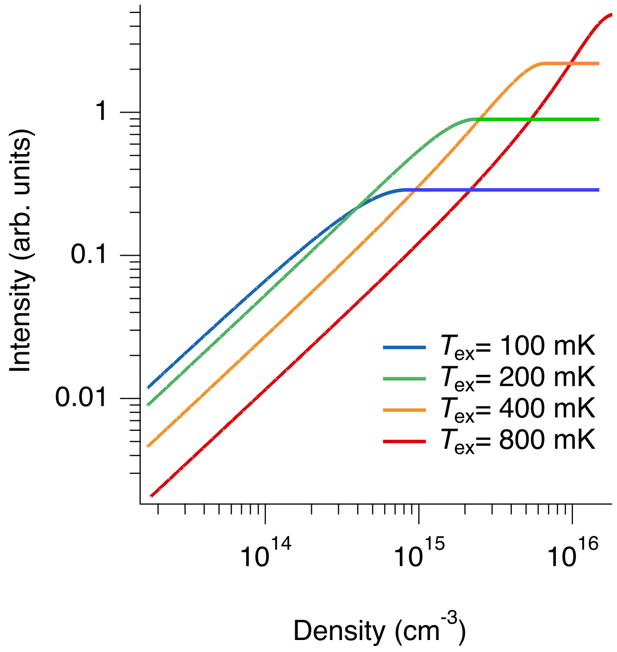

Supplementary Figure 9 | Density dependence of the calculated luminescence intensity of 1s paraexcitons at T_{ex}=100 mK (blue curve), 200 mK (green curve), 400 mK (orange curve) and 800 mK (red curve).

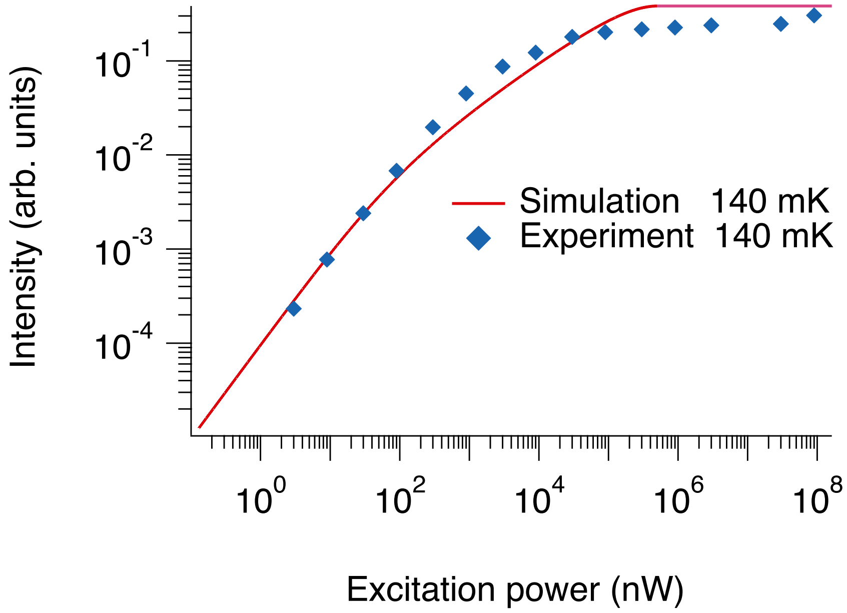

Supplementary Figure 10 | Excitation power dependence of the luminescence intensity. The red curve shows the calculated luminescence intensity at T_{ex}=140 mK. The blue diamonds show the experimental luminescence intensity at T_{ex}=140 mK. The calculated luminescence intensity is normalized to the experimental luminescence intensity at P_{pump}=30 nW.

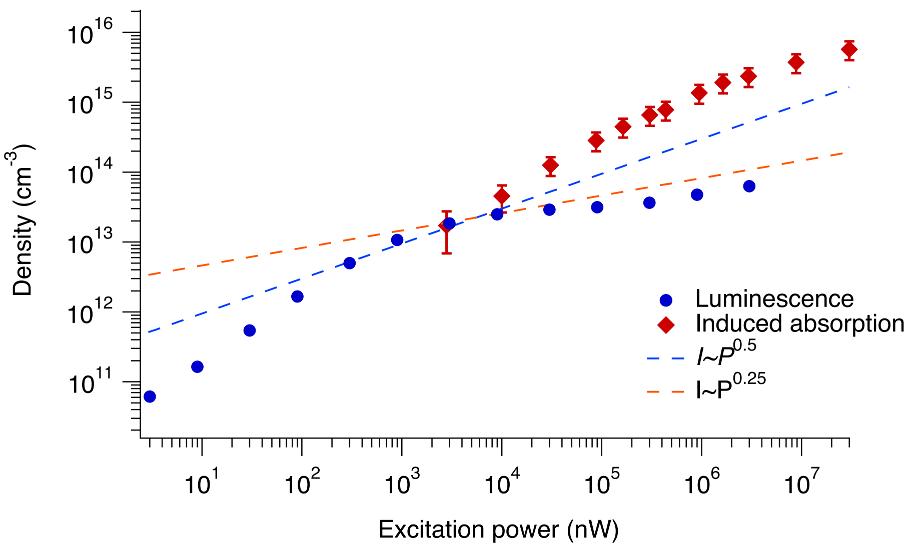

Supplementary Figure 11 | Density of trapped 1s paraexcitons as a function of excitation power. Red diamonds show absolute densities of 1s paraexcitons measured by induced absorption at various excitation powers when T_{mix} is 64 mK. The red bars show the accuracy of measuring the density of 1s paraexcitons (see the discussion in Supplementary Note 5-e.) Blue circles show densities of 1s paraexcitons estimated solely from the luminescence intensity at various excitation powers. The dashed blue (orange) line shows the excitation power dependence proportional to the 0.5th (0.25th) power of the temperature.

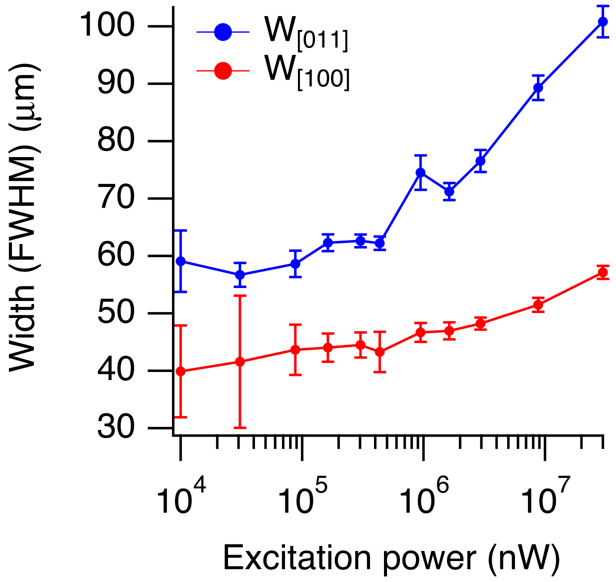

Supplementary Figure 12 | Width of the exciton cloud as a function of excitation power along the [011] crystal axis (blue curve) and the [100] crystal axis (red curve). The blue (red) bars show the statistical error of estimating the spatial width of the entire trapped paraexitons along the [011] ([100]) crystal axis (see the discussion in Supplementary Note 5-f).

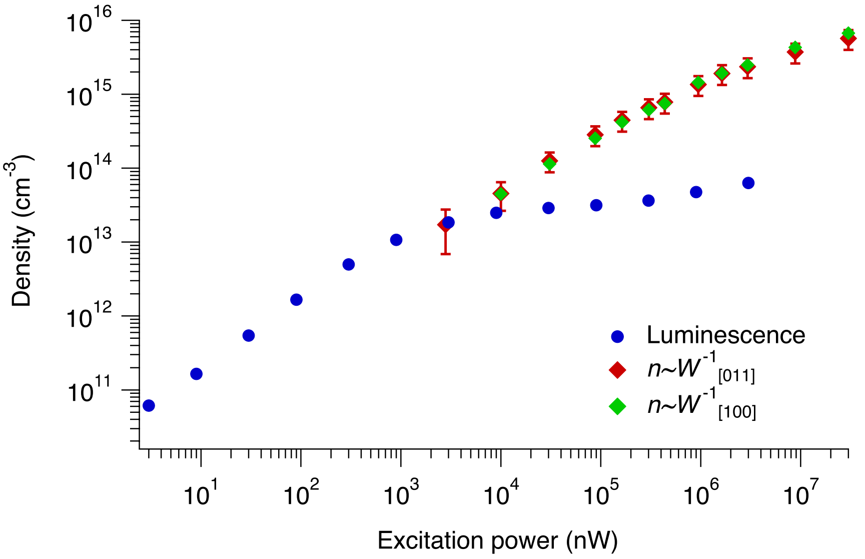

Supplementary Figure 13 | Density of trapped 1s paraexcitons at the bottom of the trap potential as a function of excitation power. Red diamonds show estimated densities of 1s paraexcitons based on the density distribution along the [011] crystal axis measured by induced absorption at various excitation powers. The red bars show the accuracy of measuring the density of 1s paraexcitons (see the discussion in Supplementary Note 5-e.) Green circles show estimated densities of 1s paraexcitons based on the density distribution along the [100] crystal axis measured by induced absorption at various excitation powers. Blue circles show densities of 1s paraexcitons estimated solely from the luminescence intensity at various excitation powers.

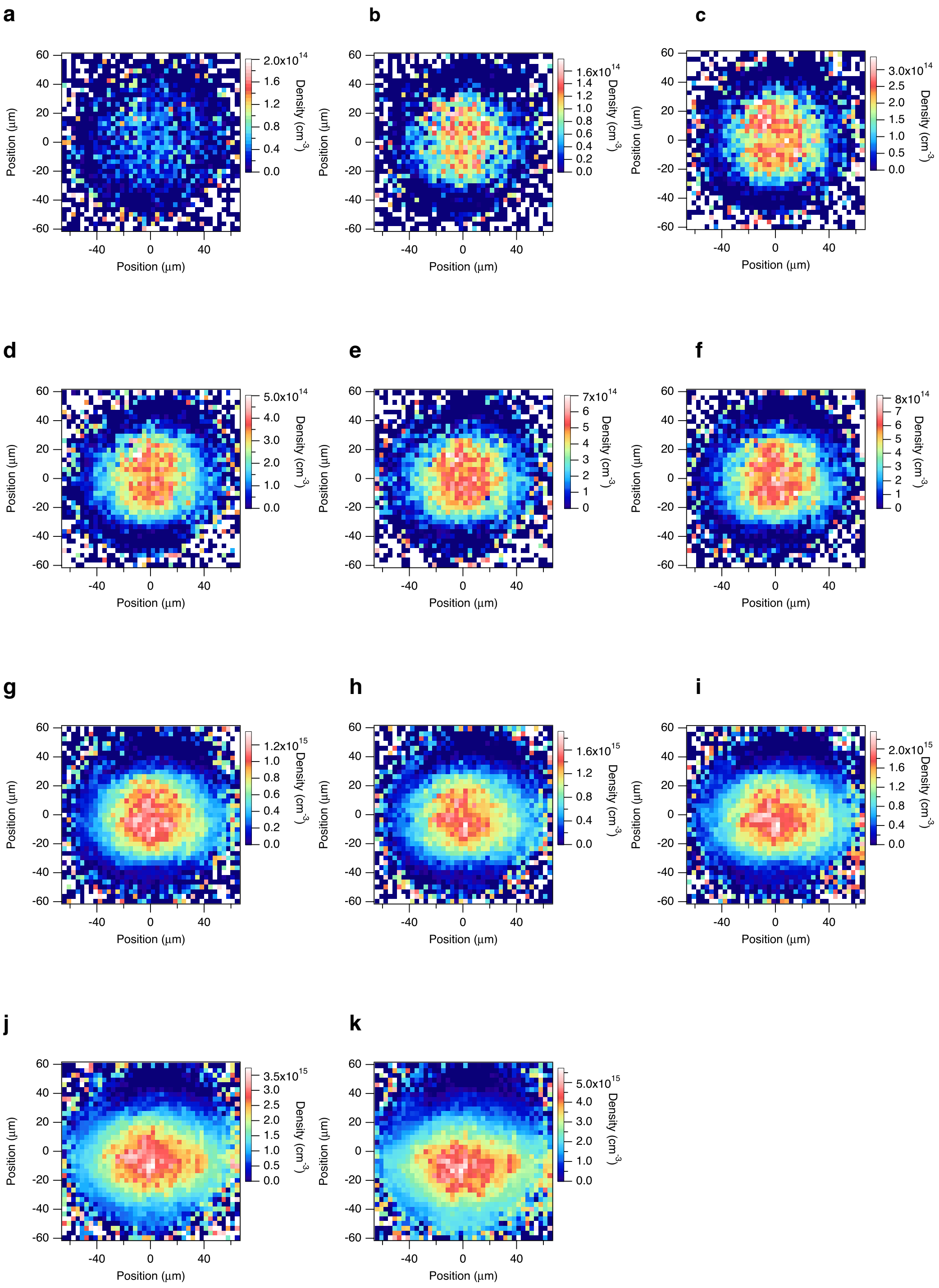

Supplementary Figure 14 l Spatial distribution of the paraexciton density measured by induced absorption imaging at T_{mix}= 64 mK for different excitation powers. (**a**) P_{pump}=10 μW, (**b**) P_{pump}=30 μW, (**c**) P_{pump}= 88 μW, (**d**) P_{pump} =160 μW, (**e**) P_{pump} =300 μW, (**f**) P_{pump} =440 μW, (**g**) P_{pump} =940 μW, (**h**) P_{pump} = 1.6 mW, (**i**) P_{pump} =2.9 mW, (**j**) P_{pump} =8.8 mW and (**k**) P_{pump} = 30 mW. These images show that a locally condensed signal appears around the centre of the cloud at P_{pump} =1.6 mW, 2.9 mW, 8.8 mW and 30 mW. The signal strongly suggests the appearance of a Bose-Einstein condensate of paraexcitons, as discussed in the main text.

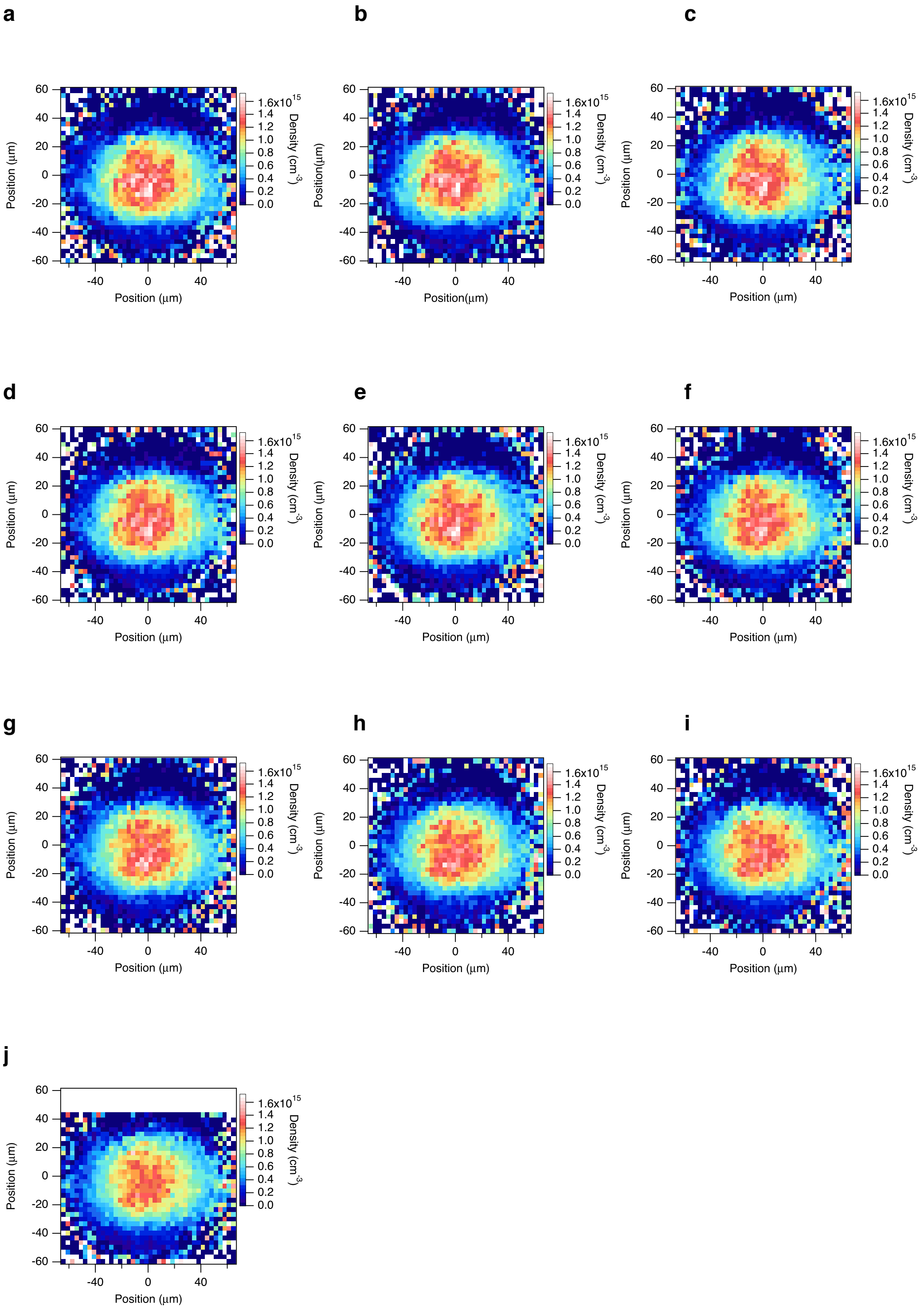

Supplementary Figure 15 I Spatial distribution of the paraexciton density measured by induced absorption imaging at P_{pump}=1.6 mW for different temperatures of the mixing chamber. (**a**) T_{mix}=100 mK, (**b**) T_{mix} =150 mK, (**c**) T_{mix} =200 mK, (**d**) T_{mix} =250 mK, (**e**) T_{mix} =300 mK, (**f**) T_{mix} =350 mK, (**g**) T_{mix} =400 mK, (**h**) T_{mix} =450 mK, (**i**) T_{mix} =500 mK and (**j**) T_{mix} =550 mK. No localized dense signal is observed at T_{mix} = 450 mK, 500 mK and 550 mK. As discussed in the main text, the threshold-like temperature dependence of the locally condensed signal strongly supports that the localized dense signal corresponds to the paraexciton condensate.

Supplementary Table

a

Data	Chi-squared value
P_{pump}=30 mW (Fig.4d)	19.4
P_{pump}=8.8 mW (Fig.4d)	26.1
P_{pump}=2.9 mW (Fig.4d)	20.6
P_{pump}=1.6 mW (Fig.4d)	20.5
T_{mix}=100 mK (Fig.4h)	24.9
T_{mix}=64 mK (Fig.4i)	22.6

b

Data	Chi-squared value
P_{pump}=30 mW (Fig.4d)	38.7
P_{pump}=8.8 mW (Fig.4d)	77
P_{pump}=2.9 mW (Fig.4d)	40.5
P_{pump}=1.6 mW (Fig.4d)	26
T_{mix}=100 mK (Fig.4h)	84
T_{mix}=64 mK (Fig.4i)	110

Supplementary Table 1 | Chi-squared test of the data shown in Figs. 4.

(a) Chi-squared value of fittings when we used a fitting function ($n(r) = \alpha \cdot \sqrt{1-\beta r^2} \cdot H(1-\beta r^2) + \gamma \cdot \exp\,[-r^2/\delta^2]$).

(b) Chi-squared value of fittings when we used a fitting function ($n(r) = \gamma \cdot \exp\,[-r^2/\delta^2]$).

Supplementary Information
"Observation of Bose-Einstein condensates of excitons in a bulk semiconductor"

Supplementary Notes

Supplementary Note 1. 1s-2p induced absorption spectrum

As explained in the main text, the 1s-2p induced absorption spectrum must be measured to determine the absolute density of 1s paraexcitons. We measured the induced absorption spectrum in a wavelength range between 9750 nm and 9940 nm, which corresponds to photon energies between 127.1 meV and 124.7 meV. The red curve in Supplementary Fig. 2 shows the intensity of the probe light that passes through windows and the crystal. As shown in Supplementary Fig. 2, Peaks of the spectral modulation appear at 9879.0 nm, 9869.7 nm, 9859.5 nm, 9837.7 nm, 9828.1 nm, 9815.5 nm, 9802.8 nm and 9788.6 nm. The fringe with a spacing of about 1×10^1 nm suggests an interference like the Fabry-Perot interference of a solid etalon with an optical length of 5 mm. The bandpass filter that we attached to the radiation shield of the refrigerator may result in this interference. The blue curve shows the differential transmission spectrum corresponding to the 1s-2p induced absorption spectrum of 1s paraexcitons at the bottom of the trap potential. The subtraction procedure associated with measurements of the differential transmission cancels out the fringe in the transmitted spectrum of the probe light.

The induced absorption spectrum has a peak at a photon energy of 126.2 meV. We performed absorption imaging at 126.2 meV. The measured spectral width (FWHM) is 1.25 meV. The measured differential transmission outside the energy range of 125.5 meV to 126.6 meV is not reliable because the narrow bandpass filters strongly attenuate the probe light when the probe wavelengths are outside the transmission range.

Supplementary Note 2. Paraexciton temperature

The paraexciton temperature is one of the most important parameters for the BEC criteria. The incoming probe light may cause a finite temperature rise. We measured the paraexciton temperature using luminescence spectroscopy to quantitatively evaluate the increase in the temperature induced by the probe light. The spectral width of the luminescence of 1s paraexcitons depends on the exciton temperature and energy resolution. When the slit width was 30 μm, the energy resolution was 50 μeV, and we observed the resolution-limited spectral width. To improve the energy resolution, we used a solid etalon (R = 90%, FSR = 648 μeV) through which collected transmitted photons, as we demonstrated in Supplementary Ref. 1. We obtained the luminescence spectrum with a high resolution of 22 μeV by scanning the angle of the solid etalon.

We performed numerical simulations that reproduce spatially integrated luminescence spectra emitted from trapped paraexcitons via the direct recombination process, taking into account the energy resolution of our measurements of the luminescence spectrum. We fit numerical simulations to each luminescence spectrum using the exciton temperature as a fitting parameter. We determined the temperature that best reproduces the spectrum as the exciton temperature. We performed numerical simulations in two steps. The first step is a theoretical prediction of the luminescence spectrum emitted from trapped paraexcitons via the direct recombination process. The weak coupling between trapped paraexcitons and the radiation field under

the strain field with minimal perturbation to the paraexciton system allows us to access to the paraexciton system with negligible perturbation. The second step is a convolution of the luminescence spectrum with a calibration function corresponding to the energy resolution of our measurements of the luminescence spectrum.

Theoretical calculations of the luminescence spectrum are based on the following equation shown in Supplementary Ref. 2.

$$I_{\mathrm{loc}}(E, \mathbf{r}) \propto \frac{\delta(E_{\mathrm{loc}})}{\exp\left[\frac{E_{\mathrm{loc}} - \mu_{\mathrm{loc}}}{k_B T}\right] - 1}, \tag{1}$$

$$E_{\mathrm{loc}} = E - V(\mathbf{r}), \tag{2}$$

$$\mu_{\mathrm{loc}} = \mu - V(\mathbf{r}), \tag{3}$$

where E, $\mathbf{r}$, I_{loc}, $V(\mathbf{r})$, E_{loc} and μ_{loc} denote the energy of the emitted photon, the position in the trap potential, the luminescence intensity as a function of the energy and the position, the potential energy at the position, the kinetic energy of paraexcitons at the position and the local chemical potential at the position, respectively. These notations are given by Supplementary Ref. 2. Supplementary Eq. (1) is based on the assumption that the translational momentum of the emitted photon in the one-photon direct recombination process is negligible. As discussed in Supplementary Note 3, we have to consider the finite translational momentum of the emitted photon in the momentum conservation principle when calculating the luminescence spectrum. Thus, we modified Supplementary Eq. (1) and added the exciton temperature as another parameter as follows:

$$I_{\mathrm{loc}}(E, \mathbf{r}, T) \propto \frac{\delta\left(E_{\mathrm{loc}} - \frac{\hbar^2 k_{\mathrm{photon}}^2}{2m}\right)}{\exp\left[\frac{E_{\mathrm{loc}} - \mu_{\mathrm{loc}}}{k_B T}\right] - 1}, \tag{4}$$

where k_{photon} denotes the wavenumber of the emitted photon.

In our experiment, we obtained spatially integrated luminescence spectra. To reproduce each spatially integrated luminescence spectrum, we integrated the calculated luminescence spectrum spatially over a region of interest determined by our measurements of luminescence spectra.

$$I(E, T) = \int_{\Delta V} I_{\mathrm{loc}}(E, \mathbf{r}, T)\, d\mathbf{r}, \tag{5}$$

where ΔV denotes the region of interest. We observed the luminescence from 1s paraexcitons using the solid etalon and the spectrometer with a slit width of 1 mm so that we could obtain all the contributions of spatially distributed 1s paraexcitons in the trap potential.

We also calculated the stress-dependent efficiency of the paraexciton luminescence. The paraexciton luminescence efficiency is quadratically dependent on the stress at the position in the trap potential (Supplementary Ref. 3), requiring a modification of the spatial integration of the calculated luminescence

intensity shown in Supplementary Eq. (5). The typical width (FWHM) of the entire trapped paraexciton cloud at an exciton temperature of 170 mK is 39 μm. The magnitude of the inhomogeneous stress at a distance of 19.5 μm from the bottom of the trap potential is 98.5% of the magnitude of the inhomogeneous stress at the bottom of the trap potential. We could regard the stress as approximately constant in the region of the trapped paraexciton cloud. Note that the calculated luminescence intensity at the distance of 19.5 μm from the bottom of the trap potential based on Supplementary Eq. (5) is 50 % of that at the bottom of the trap potential. Therefore, the position-dependent efficiency of the luminescence from trapped paraexctions hardly modifies the spatial integration of the calculated luminescence intensity.

Second, we convolved the calculated luminescence spectrum with the calibration function corresponding to the energy resolution of our measurement of the luminescence spectrum. The spectroscopy using the solid etalon resulted in a high energy resolution of 22 μeV. The energy resolution is better than that of conventional luminescence spectroscopy using a 50-cm imaging spectrometer. The energy resolution of the spectrometer was 50 μeV. Our measurements of the luminescence spectrum using the solid etalon enabled us to estimate exciton temperatures above 90 mK. In the following, we describe our measurements of the luminescence spectrum in detail and the energy resolution.

We used the solid etalon to resolve the photon energy of the luminescence from 1s paraexcitons. The luminescence light was collimated by the objective lens set inside the refrigerator and was transferred to the outside. The light passed through the solid etalon (R = 90%, FSR = 648 μeV) set on a rotation stage. The angle at which the light passes through the etalon determines the wavelength of the transmitted light. By acquiring a series of spectra by tilting the angle of the solid etalon set on the rotation stage, we can reconstruct a high-resolution spectrum. In this experiment, we tilted the solid etalon by 0.1 degrees within the range of 5.5 degrees. Supplementary Figs. 3 shows high-resolution luminescence spectra of 1s paraexcitons at P_{pump}=90 nW and T_{mix}= 64 mK.

To obtain the angle dependence of the photon energy of the transmitted light, we measured spectra of a white light that passes through the etalon. Supplementary Fig. 4 shows spectra at angles of 30 degrees,33 degrees, 35 degrees and 37 degrees. Supplementary Fig. 5 shows that the photon energy is quadratically dependent on the angle.

To determine the energy resolution of our measurements of the luminescence spectrum using the solid etalon, we also measured the neon lamp spectrum in the same manner as measurements of the luminescence spectrum. We fitted a Lorentz function to the neon spectrum as shown in Supplementary Fig. 6 and determined the energy resolution (FWHM) to be 22$\pm$2 μeV.

Next, we convolved the Lorentz function of $g(E) = \frac{1}{(11\mu\mathrm{eV})^2+E^2}$ shown in Supplementary Fig. 6 with the calculated luminescence spectrum expressed in Supplementary Eq. (5) as follows:

$$I_{\mathrm{conv}}(E,T) = \int dE' I(E',T)\, g(E-E')\,. \tag{6}$$

We fitted a test function of I_{conv} (E, T) to each luminescence spectrum using the exciton temperature as a fitting parameter. We determined the temperature that best reproduces the luminescence spectrum as the exciton temperature. Precise evaluation of the calibration function from the neon spectrum enabled us to determine precisely the exciton temperature using numerical simulations with the convolution integral.

Measurement errors of the luminescence spectrum originate from the shot noise in measurements of the luminescence intensity and the uncertainty in the photon energy of the transmitted light. The shot noise depends on the luminescence intensity and the exposure time. The typical shot noise was 2% of the luminescence intensity. The uncertainty in the photon energy of the transmitted light is determined by the uncertainty in the angle of the rotation stage of 0.05 degrees. The typical uncertainty in the photon energy is 3 μeV. The uncertainty of the angle and the goodness of fit for the calibration function ($g(E)$) determine the uncertainty in the estimated exciton temperature of 30 mK. For example, Supplementary Fig. 3a shows the experimental luminescence spectrum and the calculated luminescence spectrum at a temperature of 170 mK that best reproduces the experimental data. The chi-squared value of the fit at a degree of freedom of 24 is 26.1. We also calculated the luminescence spectrum at a temperature of 140 mK. The chi-squared value of the fit at a degree of freedom of 24 is 26.1+1.

To confirm the validity of our estimations of the exciton temperature, we measured the exciton temperature at a higher lattice temperature of 800 mK. The exciton-LA phonon interaction at the lattice temperature of 800 mK (Supplementary Ref. 4) allows paraexcitons to reach thermal equilibrium with the lattice. The exciton temperature can be estimated to be the lattice temperature without using our numerical simulations. Supplementary Fig. 7 shows the experimental luminescence spectrum at the lattice temperature of 800 mK and the calculated luminescence spectrum at a temperature of 800 mK that best reproduces the experimental data. Therefore, we confirmed the validity of our estimations of the exciton temperature.

Supplementary Figs. 3 show two typical luminescence spectra of low-density paraexcitons at P_{pump}=90 nW and T_{mix}= 64 mK when the probe light was introduced or not. We fit numerical calculations to each luminescence spectrum using the paraexciton temperature as a fitting parameter, as demonstrated in Supplementary Ref. 4. The paraexciton temperature was 170±30 mK when the probe light was on. Additionally, we evaluated the paraexciton temperature to be 150±30 mK when the probe light was off. The two temperatures match within the error range. This suggests that heat induced by the probe light had little effect on the paraexciton temperature.

Supplementary Note 3.
Luminescence intensity at weak excitation powers: Breakdown of the vertical transition model.

Fig. 2 shows the luminescence intensity increasing as a function of excitation power at T_{mix}= 50-800 mK. The excitation power ranged from 30 nW to 90 mW. We focus on the region below P_{pump}= 900 nW in Fig. 2 where the signal increases linearly with excitation power at all temperatures. In this excitation power region, the signal intensity increases when the temperature of the mixing chamber is reduced from 800 mK to 50 mK.

As explained in the main text, we integrated spectrally over 60 μeV and spatially over 16 μm around the pixel with the peak signal intensity. The number of paraexcitons and the emission efficiency in the region of interest determine the integrated luminescence intensity. As for the paraexciton number, the average kinetic energy of trapped paraexcitons proportionally decreases when the exciton temperature is reduced, resulting in the increase in the number of paraexcitons in the region of interest. The increase in the number of paraexcitons leads to the increase in the signal intensity. On the other hand, the temperature dependence of the emission efficiency has never been studied before in the present temperature range.

To obtain the temperature dependence of the relative emission efficiency, we detected the luminescence from trapped 1s paraexcitons. We set the entrance slit of the spectrometer to 1mm so that we could obtain all the contributions of spatially distributed 1s paraexcitons in the trap potential. Opening the entrance slit to 1 mm resulted in a poor energy resolution. This brought about broadening of the measured luminescence spectrum. We integrated the signal spectrally between 2016 meV and 2021 meV to measure the luminescence intensity of paraexcitons. The peak energy of the paraexciton luminescence was at approximately 2018.2 meV. Supplementary Fig. 8 shows the integrated luminescence intensity at various temperatures with a fixed number of total trapped 1s paraexcitons. It shows that the signal increases as the exciton temperature is reduced, meaning that the increase in emission efficiency. Quantitatively, the luminescence intensity increases by 30% when the temperature decreases from 200 mK to 130 mK.

The temperature dependence is completely beyond the conventional understanding of the corresponding luminescence process. In solid-state spectroscopy, the translational momentum of the emitted photon in the one-photon direct recombination process is widely accepted to be negligible. This assumption is well known as the "vertical transition" and means that only stationary excitons can emit photons. The black dashed curve in Supplementary Fig. 8 shows the predicted signal intensity assuming the vertical transition, which is proportional to the -1.5th power of the temperature ($\sim T_{ex}^{-1.5}$) as explained in Supplementary Note 4-a. However, it deviates significantly from the experimental result at $T_{ex} \leq 400$ mK, corresponding to breakdown of the vertical transition model.

In contrast, our experimental results agree well with the theoretical calculations over the whole temperature range, as shown using the blue curve in Supplementary Fig. 8. As explained in Supplementary Note 4-a, we took into account the finite translational momentum of the emitted photon (p_0) in the momentum conservation principle when calculating the proper emission efficiency. We did not include polariton effects because 1s paraexcitons are very weakly coupled to the radiation field. The agreement between the experimental results and our calculations shows that the translational momentum of the emitted photon is non-negligible at dilution temperatures. Indeed, $p_0 = 3\times10^{-27}$ kg·m/s corresponds to the average translational momentum of the trapped paraexcitons in thermal equilibrium at $T_{ex} = 120$ mK. The agreement also suggests that the paraexciton condensate makes no contribution to the direct recombination process because the condensate has a zero-average translational momentum. Note that the change in the emission efficiency hardly affects the paraexciton lifetime because the direct recombination process is very weakly allowed.

Supplementary Note 4. Numerical simulation of the direct recombination process of 1s paraexcitons

4-a Temperature dependence of the luminescence intensity at weak excitation powers

Supplementary Fig. 8 shows the calculated luminescence intensity at various temperatures. The energy-momentum conservation law limits the paraexcitons that can participate in the direct recombination process. The luminescence intensity is approximately proportional to the total number of paraexcitons satisfying the conservation law. Note that the inhomogeneous strain field weakly allows the direct recombination process of 1s paraexcitons. Therefore, the paraexciton luminescence efficiency depends on the inhomogeneous stress. As explained at the end of this section, the position-dependent efficiency of the paraexciton luminescence hardly modifies the calculated temperature dependence of the spatially integrated luminescence intensity.

As shown in the following, we evaluated the total number of 1s paraexcitons that can recombine by the direct recombination process. We specify the 1s paraexciton state by two parameters (**r**, **p**), where **r** and **p** are the position and momentum of the paraexcitons in a trap potential. The energy-momentum conservation law requires the following equations between a paraexciton and an emitted photon to hold in the direct recombination process:

$$\frac{p^2}{2m} + V(\mathbf{r}) + E_{\mathrm{g}} - E_{\mathrm{bind}} = \hbar\omega, \tag{7}$$

$$p = \hbar\frac{n\omega_{\mathrm{photon}}}{c}\ , \tag{8}$$

where m, $V(\mathbf{r})$, E_{g}, E_{bind}, ω_{photon} and n denote the effective mass of 1s paraexcitons, trap potential at the position, band gap energy, binding energy of 1s paraexcitons, angular frequency of the photon emitted through direct recombination of paraexcitons and refractive index of cuprous oxide, respectively. We can express the momentum satisfying Supplementary Eqs. (7) and (8) as a function of position ($p_{\mathrm{emission}}(\mathbf{r})$):

$$p_{\mathrm{emission}}(\mathbf{r}) = m\cdot\left(\frac{c}{n} - \sqrt{\left(\frac{c}{n}\right)^2 - \frac{2}{m}\left(V(\mathbf{r}) + E_{\mathrm{g}} - E_{\mathrm{bind}}\right)}\right). \tag{9}$$

In practice, paraexcitons having momenta of $p_{\mathrm{emission}}(\mathbf{r})-\Delta p < p < p_{\mathrm{emission}}(\mathbf{r})+\Delta p$ can contribute to the direct recombination process. Δp is a finite value determined, for example, by the uncertainty in the momentum originating from spatial confinement of paraexcitons. Here, we treat Δp as a constant that does not depend on the position or temperature.

Using Supplementary Eq. (9), we calculate p_{emission} (**r**=0) $=3\times10^{-27}$ kg·m/s, where **r**=0 corresponds to the bottom of the trap potential. p_{emission} (**r**=0) corresponds to the equivalent kinetic energy at 120 mK:

$$\frac{p_{\mathrm{emission}}(0)^2}{2m} = 120\ \mathrm{mK}. \tag{10}$$

In contrast, the vertical transition model approximates the value of p_{emission} as zero:

$$\forall \mathbf{r}\ \ p_{\mathrm{emission}}(\mathbf{r}) \approx 0. \tag{11}$$

We calculate the number of paraexcitons that satisfy the conditions for the direct recombination process described above. The statistical distribution of trapped paraexcitons ($f(\mathbf{r}, \mathbf{p}, T_{\mathrm{ex}}, \mu)$) is expressed as

$$f(\mathbf{r}, \mathbf{p}, T_{\mathrm{ex}}, \mu) = \frac{1}{\left[\exp\left(\frac{\left(\frac{\mathbf{p}^2}{2m} + V(\mathbf{r}) - \mu\right)}{k_{\mathrm{B}}T_{\mathrm{ex}}}\right) - 1\right]}, \tag{12}$$

where T_{ex} and μ denote the temperature of the paraexcitons and the chemical potential, respectively. The chemical potential depends on the exciton temperature and the total number of paraexcitons (N) through the following equation:

$$N = \iint d\mathbf{r} \frac{d\mathbf{p}}{(2\pi\hbar)^3} f(\mathbf{r}, \mathbf{p}, T_{\mathrm{ex}}, \mu). \tag{13}$$

Therefore, we can express the chemical potential as a function of temperature and total number of paraexcitons ($\mu(T_{\mathrm{ex}}, N)$) using Supplementary Eq. (13).

The luminescence intensity as a function of position, exciton temperature and total number is expressed as

$$I(\mathbf{r}, T_{\mathrm{ex}}, N) \propto f\left(\mathbf{r}, \mathbf{p}|_{p=p_{\mathrm{emission}}(\mathbf{r})}, T_{\mathrm{ex}}, \mu(T_{\mathrm{ex}}, N)\right) \cdot \Delta p\,. \tag{14}$$

The spatially integrated luminescence intensity is also evaluated as

$$I(\mathbf{r}, T_{\mathrm{ex}}, N) \propto \int d\mathbf{r}\ f\left(\mathbf{r}, \mathbf{p}|_{p=p_{\mathrm{emission}}(\mathbf{r})}, T_{\mathrm{ex}}, \mu(T_{\mathrm{ex}}, N)\right) \cdot \Delta p\,. \tag{15}$$

As shown in Supplementary Fig. 8, we measured the luminescence intensity at various temperatures for P_{pump} =90 nW. The density of paraexcitons was well below the quantum degenerate regime. Therefore, we can approximate Supplementary Eq. (12) as

$$f(\mathbf{r}, \mathbf{p}, T_{\mathrm{ex}}, \mu) \simeq \exp\left(\frac{\mu}{k_{\mathrm{B}}T_{\mathrm{ex}}}\right) \cdot \exp\left(-\frac{\frac{\mathbf{p}^2}{2m} + V(\mathbf{r})}{k_{\mathrm{B}}T_{\mathrm{ex}}}\right). \tag{16}$$

We substitute this function into Supplementary Eq. (13) as follows:

$$N = \iint d\mathbf{r} \frac{d\mathbf{p}}{(2\pi\hbar)^3}\ f(\mathbf{r}, \mathbf{p}, T_{\mathrm{ex}}, \mu) \simeq \exp\left(\frac{\mu}{k_{\mathrm{B}}T_{\mathrm{ex}}}\right) \cdot \left(\frac{k_{\mathrm{B}}T_{\mathrm{ex}}}{\hbar\omega}\right)^3, \tag{17}$$

where ω denotes the trap frequency. As a result, the statistical distribution as a function of position, momentum, temperature, and total number is denoted

$$f(\mathbf{r}, \mathbf{p}, T_{\mathrm{ex}}, N) \simeq N \cdot \left(\frac{\hbar\omega}{k_{\mathrm{B}}T_{\mathrm{ex}}}\right)^3 \cdot \exp\left(-\frac{\frac{\mathbf{p}^2}{2m} + V(\mathbf{r})}{k_{\mathrm{B}}T_{\mathrm{ex}}}\right). \quad (18)$$

We calculate the spatially integrated luminescence intensity as a function of temperature and total number:

$$I(T_{\mathrm{ex}}, N) \propto \int d\mathbf{r}\, f(\mathbf{r}, \mathbf{p}|_{p=p_{\mathrm{emission}}(\mathbf{r})}, T_{\mathrm{ex}}, \mu(T_{\mathrm{ex}}, N)) \cdot \Delta p$$

$$\simeq \int d\mathbf{r}\; N \cdot \left(\frac{\hbar\omega}{k_{\mathrm{B}}T_{\mathrm{ex}}}\right)^3 \cdot \exp\left(-\frac{\frac{p_{\mathrm{emission}}(\mathbf{r})^2}{2m} + V(r)}{k_{\mathrm{B}}T_{\mathrm{ex}}}\right) \cdot \Delta p. \quad (19)$$

For comparison, we calculate the spatially integrated luminescence intensity based on the vertical transition model. We substitute Supplementary Eq. (11) into Supplementary Eq. (19):

$$I(T_{\mathrm{ex}}, N) \propto \int d\mathbf{r}\; f\left(\mathbf{r}, \mathbf{p}|_{p=p_{\mathrm{emission}}(\mathbf{r})}, T_{\mathrm{ex}}, \mu(T_{\mathrm{ex}}, N)\right) \cdot \Delta p$$

$$\simeq \int d\mathbf{r}\; N \cdot \left(\frac{\hbar\omega}{k_{\mathrm{B}}T_{\mathrm{ex}}}\right)^3 \cdot \exp\left(-\frac{\frac{p_{\mathrm{emission}}(\mathbf{r})^2}{2m} + V(\mathbf{r})}{k_{\mathrm{B}}T_{\mathrm{ex}}}\right) \cdot \Delta p$$

$$\simeq \int d\mathbf{r}\, N \cdot \left(\frac{\hbar\omega}{k_{\mathrm{B}}T_{\mathrm{ex}}}\right)^3 \cdot \exp\left(-\frac{V(\mathbf{r})}{k_{\mathrm{B}}T_{\mathrm{ex}}}\right) \cdot \Delta p$$

$$= N \cdot \left(\frac{2\pi\hbar^2}{k_{\mathrm{B}}T_{\mathrm{ex}}}\right)^{1.5} \cdot \Delta p. \quad (20)$$

Therefore, the calculated luminescence intensity based on the vertical transition model depends on the -1.5th power of the temperature.

We also calculate the luminescence intensity taking into account the stress-dependent efficiency of the paraexciton luminescence. The paraexciton luminescence efficiency is quadratically dependent on the stress at the position in the trap potential (Supplementary Ref. 3). Therefore, the spatially integrated luminescence intensity is evaluated by extending Supplementary Eq. (19) as

$$I(T_{\mathrm{ex}}, N) \propto \int d\mathbf{r}\; \sigma^2(\mathbf{r}) \cdot f\left(\mathbf{r}, \mathbf{p}|_{p=p_{\mathrm{emission}}(\mathbf{r})}, T_{\mathrm{ex}}, \mu(T_{\mathrm{ex}}, N)\right) \cdot \Delta p$$

$$\simeq \int d\mathbf{r}\, N \cdot (\hbar\omega/k_{\mathrm{B}}T_{\mathrm{ex}})^3 \cdot \sigma^2(\mathbf{r}) \cdot \exp\left(-\frac{\frac{p_{\mathrm{emission}}(\mathbf{r})^2}{2m} + V(\mathbf{r})}{k_{\mathrm{B}}T_{\mathrm{ex}}}\right) \cdot \Delta p, \quad (21)$$

Where $\sigma(\mathbf{r})$ denotes the stress as a function of position. Our calculation shows that the luminescence intensity increases by 38.4% (38.1%) according to eq. Supplementary Eq. (21) (Supplementary Eq. (19)) when the temperature decreases from 200 mK to 130 mK. Therefore, the stress-dependent efficiency of the

paraexciton luminescence has little effect on the temperature dependence of the spatially integrated luminescence intensity.

4-b Luminescence intensity in a quantum degenerate regime

We calculate the luminescence intensity and density of 1s paraexcitons at the bottom of the trap potential at various chemical potentials using Supplementary Eq. (14) and the following equation:

$$n(\mathbf{r}=0, T_{\mathrm{ex}}, \mu) = \int d\mathbf{p}\, f(\mathbf{r}=0, \mathbf{p}, T_{\mathrm{ex}}, \mu)\,. \tag{22}$$

Supplementary Fig. 9 shows the calculated density dependence of the luminescence intensity at various temperatures. Supplementary Fig. 9 possesses the following two features.

1. The luminescence intensity decreases by 85% when the exciton temperature is increased from 100 mK to 800 mK at low densities below 10^{14} cm^{-3}. This occurs because a larger number of paraexcitons occupy greater momentum states than p_{emission} ($\mathbf{r}$=0) at higher temperatures.
2. Conversely, the luminescence intensity decreases by 95% when the exciton temperature is reduced from 800 mK to 100 mK at high densities above 8×10^{15} cm^{-3}. The reduction in temperature results in a lower paraexciton density that is required for the transition to the quantum statistical regime. As discussed in the main text, the transition to the quantum degenerate regime results in saturation of the luminescence intensity. In the classical regime, conversely, the luminescence intensity is simply proportional to the total number of 1s paraexcitons.

4-c Excitation power dependence of the luminescence intensity

Fig. 2 shows the excitation power dependence of the luminescence intensity at T_{mix}=50 mK. When we measured the luminescence intensity, we confirmed that the paraexciton temperature was 140±30 mK. In the main text, we concluded that the saturation of the luminescence intensity at P_{pump}> 9 μW suggests a transition to a quantum degenerate regime. Here, we calculate the excitation power dependence of the luminescence intensity at T_{ex}=140 mK to theoretically support this conclusion.

First, we estimate the paraexciton density as a function of excitation power, and then, we calculate the density-dependent luminescence intensity as discussed in Supplementary Note 4-b. The rate equation for the position-dependent density of 1s paraexcitons in the trap potential is given as follows: We set a simple rate equation between the position-dependent paraexciton density at the bottom of the trap potential and the excitation power:

$$\frac{d\,n(\mathbf{r})}{dt} = -\frac{n(\mathbf{r})}{\tau} - An(\mathbf{r})^2 + g\big(P_{\mathrm{pump}}, \mathbf{r}\big), \tag{23}$$

where τ, A and $g(P_{\mathrm{pump}}, \mathbf{r})$ denote the lifetime of paraexcitons, coefficient of the two-body collision-induced loss (set to 10^{-16} cm^3/ns (Supplementary Ref. 5)) and generation rate of paraexcitons at the position as a function of excitation power, respectively. Note that the lifetime of paraexcitons is much shorter than the radiative lifetime due to the density-independent non-radiative recombination of paraexcitons. The radiative lifetime of paraexcitons is reported[25] to be 7 ms when no stress is applied to the sample and is dominated by the phonon-assisted radiative recombination, while direct recombination is forbidden. Although the radiative lifetime becomes shorter because the direct recombination process is activated (and increases quadratically on the applied stress), the radiative lifetime is much longer than the density-independent non-radiative lifetime ($\tau_{\mathrm{background}}$ =600 ns), and is maintained at all positions in the strain-induced trap potential. Therefore, the lifetime (τ) is expressed as

$$\tau \approx \tau_{\mathrm{background}} \,. \tag{24}$$

Based on Supplementary Eqs. (23) and (24), the paraexciton density in the steady state (n_{s}) is

$$n_{\mathrm{s}}(\mathbf{r}) = \frac{\sqrt{1 + 4A\tau_{\mathrm{background}}{}^2 g(P_{\mathrm{pump}}, \mathbf{r})} - 1}{2A\tau_{\mathrm{background}}} \,. \tag{25}$$

Therefore, the generation rate and the two-body collision-induced loss determine the paraexciton density at various excitation powers. The position-dependent generation rate of paraexcitons satisfies the following equation:

$$\int d\mathbf{r}\; g(P_{\mathrm{pump}}, \mathbf{r}) = G(P_{\mathrm{pump}}), \tag{26}$$

where $G(P_{\mathrm{pump}})$ denotes spatially integrated generation rate of paraexcitons in the trap potential as a function of excitation power.

The spatially integrated generation rate of paraexcitons in the trap potential was experimentally determined by the absorption ratio of the excitation light and the ratio of the number of trapped paraexcitons to that of all the generated orthoexcitons, which is called “the trap efficiency”. We experimentally confirmed that the absorption ratio was 50%. We already observed that paraexcitons become diffusive at dilution temperatures and that impurity scattering and local potential fluctuations have little effect on the trapping of paraexcitons (Supplementary Ref. 6). Based on our findings, we assumed that all the generated orthoexcitons were converted to trapped paraexcitons. Therefore, the spatially integrated generation rate of paraexcitons in the trap potential can be expressed as

$$G(P_{\mathrm{pump}}) = 0.5 \cdot \frac{P_{\mathrm{pump}}}{\hbar\omega_{\mathrm{photon}}}, \tag{27}$$

where ω_{photon} denotes the angular frequency of the excitation light. We substitute Supplementary Eq. (27) into Supplementary Eq. (26):

$$\int dr\; g(P_{\mathrm{pump}}, r) = 0.5 \cdot \frac{P_{\mathrm{pump}}}{\hbar\omega_{\mathrm{photon}}}. \tag{28}$$

Supplementary Eq. (28) determines the excitation power dependence of the position-dependent generation rate of paraexcitons.

Using Supplementary Eq. (25) at low excitation powers, we can approximate the position-dependent generation rate as

$$g(P_{\text{pump}}, \mathbf{r}) \simeq \frac{n_{\text{s}}(\mathbf{r})}{\tau_{\text{background}}}. \tag{29}$$

Furthermore, the spatial distribution of the paraexciton density at low densities is determined by the paraexciton temperature and the total number of paraexcitons based on Supplementary Eq. (18):

$$n_{\text{s}}(\mathbf{r}) = \int d\mathbf{p}\, f(\mathbf{r}, \mathbf{p}, T_{\text{ex}}, N) \simeq N \cdot (\hbar\omega/k_{\text{B}}T_{\text{ex}})^3 \cdot \int d\mathbf{p} \exp\left(-\frac{\frac{\mathbf{p}^2}{2m} + V(\mathbf{r})}{k_{\text{B}}T_{\text{ex}}}\right)$$

$$= N \cdot (\hbar\omega/k_{\text{B}}T_{\text{ex}})^3 \cdot (\sqrt{2\pi m k_{\text{B}}T_{\text{ex}}})^3 \cdot \exp\left(-\frac{V(\mathbf{r})}{k_{\text{B}}T_{\text{ex}}}\right), \tag{30}$$

where N denotes the total number of paraexcitons as a function of excitation power. Therefore, the position dependence of the generation rate is expressed as:

$$g(P_{\text{pump}}, \mathbf{r}) \sim \exp\left(-\frac{V(\mathbf{r})}{k_{\text{B}}T_{\text{ex}}}\right). \tag{31}$$

As a result, we obtain the following equation using Supplementary Eqs. (28) and (31):

$$g(P_{\text{pump}}, \mathbf{r}) = 0.5 \cdot \frac{P_{\text{pump}}}{\hbar\omega_{photon}} \cdot \left(\frac{\hbar\omega}{k_{\text{B}}T_{\text{ex}}}\right)^3 \cdot \left(\sqrt{2\pi m k_{\text{B}}T_{\text{ex}}}\right)^3 \cdot \exp\left(-\frac{V(\mathbf{r})}{k_{\text{B}}T_{\text{ex}}}\right). \tag{32}$$

Note that, in principle, the position-dependent generation rate around the bottom of the trap potential is determined by the transport of paraexcitons from the excitation position to the bottom of the trap potential. We observed that paraexciton transport enters a ballistic regime at dilution temperatures[55]. The excitation beam was focused on the region containing the bottom of the trap potential. Therefore, the transport process of paraexcitons from the excitation region to the bottom of the trap potential is completed rapidly. The loss of paraexcitons due to the two-body inelastic collision during the transport process is negligibly small even at the BEC transition density. Therefore, we safely assume that Supplementary Eq. (32) holds at various excitation powers.

We substitute Supplementary Eq. (32) into Supplementary Eq. (25):

$$n_{\text{s}}(\boldsymbol{r}) = \frac{\sqrt{1 + 2A\tau_{\text{background}}{}^2 \cdot \frac{1}{\hbar\omega_{\text{photon}}} \cdot \left(\frac{\hbar\omega}{k_{\text{B}}T_{\text{ex}}}\right)^3 \cdot \left(\sqrt{2\pi m k_{\text{B}}T_{\text{ex}}}\right)^3 \cdot \exp\left(-\frac{V(\boldsymbol{r})}{k_{\text{B}}T_{\text{ex}}}\right) \cdot P_{\text{pump}}} - 1}{2A\tau_{\text{background}}} \tag{33}$$

Supplementary Eq. (33) gives the relation between the paraexciton density at the bottom of the trap potential and the excitation power, taking into account the two-body collision-induced loss. Furthermore, we calculate the density dependence of the luminescence intensity at T_{ex}=140 mK based on Supplementary Eqs. (14) and

(22). Supplementary Fig. 10 shows that the numerical calculation well reproduces the strong saturation of the luminescence that occurs at P_{pump}> 9 μW. Therefore, this saturation suggests a transition to the quantum degenerate regime, as discussed in Supplementary Note 3.

4-d Strong saturation of the luminescence intensity

As explained in the main text, Fig. 2 shows a strong saturation of the intensity at P_{pump}> 30 μW that has never been observed when the temperature of the mixing chamber is 50 mK. The red dashed line shows the strongest saturation previously reported ($I \propto P_{\mathrm{pump}}^{0.25}$). This 0.25 power dependence can be found when a high-power excitation takes place near the rim of the trap, and when the temperature is above 1 K, i.e. the mobility of paraexcitons is lower (neither condition applies to our experiment). The power dependence is attributed to the combined effect of the two-body inelastic loss of paraexcitons accumulated in the trap and the fact that the two-body inelastic loss also occurs during the migration of the trap potential to lower energy positions. The saturation of the intensity at P> 30 μW is stronger than that the red dashed line shows.

Fig. 3 also shows a strong saturation of the luminescence intensity at P_{pump}> 30 μW. Supplementary Fig. 11 shows the densities of trapped 1s paraexcitons at various excitation powers which are the same as the data shown in Fig. 3. The orange dashed line in Supplementary Fig. 11 shows the strongest saturation previously reported ($I \propto P_{\mathrm{pump}}^{0.25}$). The saturation of the intensity at P_{pump}> 30 μW is stronger than that the orange dashed line shows. The absolute density at P_{pump}=90 μW, where the strong saturation occurs, is 2.8×10^{14} cm^{-3}. The absolute density of 2.8×10^{14} cm^{-3} is 16% of the transition density, suggesting the transition to the quantum degenerate regime. Therefore, the strong saturation suggests the transition to the quantum degenerate regime.

Supplementary Note 5. Measurements of the paraexciton density

5-a Estimation of the paraexciton density using luminescence spectroscopy

When the approximation in Supplementary Eq. (16) holds, the luminescence intensity is proportional to the density. We define the luminescence intensity at the bottom of the trap potential at P_{pump}= 90 nW as

$$I\left(P_{\mathrm{pump}} = 90\ \mathrm{nW}\right) = \alpha \cdot n\left(P_{\mathrm{pump}} = 90\ \mathrm{nW}\right), \tag{34}$$

where α denotes a coefficient that relates the luminescence intensity and the paraexciton density.
Using Supplementary Eq. (30), we calculate the paraexciton density at the bottom of the trap potential and determine α. Therefore, we can estimate the density at other excitation powers in the linear regime using alpha as follows:

$$n\left(P_{\mathrm{pump}}\right) = \alpha^{-1} \cdot I\left(P_{\mathrm{pump}}\right). \tag{35}$$

The blue circles in Fig. 3 show the paraexciton density estimated as explained in this section.

5-b Measurement of the absolute density using the induced absorption

The equation between the induced absorption coefficient and the 1s paraexciton density is expressed based on the Lorentz oscillator model as

$$n_{1s} = \frac{\hbar \varepsilon c n_{\mathrm{B}}}{\pi |\mu_{1\mathrm{s}-2\mathrm{p}}|^2 E_{1\mathrm{s}-2\mathrm{p}}} \int \Delta\alpha_{1\mathrm{s}-2\mathrm{p}}(E) dE, \tag{36}$$

where ε, n_{B}, $\mu_{1\mathrm{s}\text{-}2\mathrm{p}}$, $E_{1\mathrm{s}\text{-}2\mathrm{p}}$, E and $\Delta\alpha_{1\mathrm{s}\text{-}2\mathrm{p}}$ (E) are the dielectric constant, refractive index of cuprous oxide, energy level interval between the 1s state and 2p state, photon energy of the probe light and induced absorption coefficient, respectively.

The induced absorption coefficient depends on the position because the spatial distribution of paraexcitons is inhomogeneous. When the probe light propagates along the $[01\bar{1}]$ crystal axis, the intensity of the probe light is expressed as

$$\frac{dJ(x, y, z, E)}{dy} = -\{\alpha_0(E) + \Delta\alpha(x, y, z, E)\} \cdot J(x, y, z, E), \tag{37}$$

where x, y, z, $J(x,y,z,E)$, α_0 and $\Delta\alpha$ denote the position along the $[011]$ crystal axis, position along the $[01\bar{1}]$ crystal axis, position along the $[100]$ crystal axis, intensity of the probe light as a function of position and photon energy E, absorption coefficient of cuprous oxide without paraexcitons at E and induced absorption coefficient at E, respectively.

We measured the intensity of the probe light when the excitation light was on/off. We can describe the intensities using Supplementary Eq. (37) as

$$\frac{J_1(x, z, E)}{J_0(x, z, E)} = \exp\left[\int -\alpha(x, y, z, E)\, dy\right], \tag{38}$$

$$\frac{J_2(x, z, E)}{J_0(x, z, E)} = \exp\left[\int -\alpha(x, y, z, E)\, dy + \int -\Delta\alpha(x, y, z, E)\, dy\right], \tag{39}$$

where J_0, J_1, and J_2 denote the intensity of the probe light before passing through the crystal, after passing through the crystal without the excitation light and after passing through the crystal with the excitation light, respectively. We experimentally obtained the differential transmission as

$$\frac{J_1(x,z,E) - J_2(x,z,E)}{J_1(x,z,E)} = 1 - \exp\left[\int -\Delta\alpha(x, y, z, E)\, dy\right] \simeq \int \Delta\alpha(x, y, z, E)\, dy. \tag{40}$$

We chose a photon energy of 126.2 meV for the absorption imaging measurements. As mentioned in (2-c), we experimentally obtained the induced absorption spectrum and determined a conversion coefficient (β) that relates the spectral integration of the absorption coefficient spectrum and the absorption coefficient at 126.2 meV, which is important to evaluate the paraexciton density:

$$\beta = \frac{1}{\Delta\alpha(E = 126.2\ \mathrm{meV})} \cdot \int \alpha(E) dE\,. \tag{41}$$

As a result, we obtain the following equation using Supplementary Eq. (36), (40) and (41):

$$\frac{J_1(x,z,E=126.2\ \mathrm{meV})-J_2(x,z,E=126.2\ \mathrm{meV})}{J_1(x,z,E=126.2\ \mathrm{meV})}=[\frac{\hbar\varepsilon c n_\mathrm{B}}{\pi|\mu_{1\mathrm{s}-2\mathrm{p}}|^2E_{1\mathrm{s}-2\mathrm{p}}}]^{-1}\cdot\beta\int n_{1\mathrm{s}}(x,y,z)dy\,. \quad (42)$$

We evaluated the y-direction spatially integrated density of paraexcitons from the differential transmission using Supplementary Eq. (42). However, we needed to know the density distribution along the y-axis to determine the paraexciton density at the bottom of the trap potential. As shown in Supplementary Eq. (12), the density distribution depends on the trap potential. Here, the trap frequency along the $[01\bar{1}]$ crystal axis is the same as that along the $[011]$ crystal axis (see the discussion in Supplementary Note 5-f). Absorption images show the distribution of the differential transmission along the x-axis. When the paraexciton gas did not satisfy the BEC criteria, we used Gaussian fitting:

$$\frac{J_1\,(x,z=0,E)-J_2(x,0,E)}{J_1(x,z=0,E)}\simeq\frac{J_1(x=0,z=0,E)-J_2(x=0,z=0,E)}{J_1(x=0,z=0,E)}\cdot\exp\left[-\frac{x^2}{{W_\mathrm{fit}}^2}\right]. \quad (43)$$

Using a fitting parameter of W_fit, we approximate Supplementary Eq. (42) as

$$\frac{J_1\,(x,z=0,E)-J_2(x,0,E)}{J_1(x,z=0,E)}\simeq[\frac{\hbar\varepsilon c n_\mathrm{B}}{\pi|\mu_{1\mathrm{s}-2\mathrm{p}}|^2E_{1\mathrm{s}-2\mathrm{p}}}]^{-1}\cdot\beta\cdot\int n_{1\mathrm{s}}(x,y=0,z)\cdot\exp\left[-\frac{y^2}{{W_\mathrm{fit}}^2}\right]dy$$

$$=[\frac{\hbar\varepsilon c n_\mathrm{B}}{\pi|\mu_{1\mathrm{s}-2\mathrm{p}}|^2E_{1\mathrm{s}-2\mathrm{p}}}]^{-1}\cdot\beta\cdot\sqrt{\pi{W_\mathrm{fit}}^2}\cdot n_{1\mathrm{s}}(x,y=0,z). \quad (44)$$

Based on Supplementary Eq. (44), we evaluated the absolute density of 1s paraexcitons at the trap bottom (x=0, y=0, z=0) and at other positions in the x-z plane (x, y=0, z) shown in Fig. 3 and Figs. 4. Therefore, the spatial distribution of the paraexciton density shown in Figs. 4 is essentially the same as the original data, which are the spatially resolved differential transmission. Note that we did not assume that a paraexciton condensate appears in Supplementary Eq. (44).

5-c Paraexciton density at the bottom of the trap potential

Measurement of the paraexciton density based on Supplementary Eq. (43) does not hold when the condensate appears because the density distribution remarkably deviates from the Gaussian distribution. The density distribution is described as the summation of the thermal component and the condensate:

$$n_{1\mathrm{s}}(x,y=0,z)=n_0\cdot\exp\left[-4\ln2\cdot\frac{x^2}{{W_\mathrm{x}}^2}\right]+n_\mathrm{c}\left(1-2\frac{x^2}{{W_\mathrm{xc}}^2}\right)\cdot H\left(1-2\frac{x^2}{{W_\mathrm{xc}}^2}\right), \quad (45)$$

where n_0, W_x, n_c, W_xc, and H denote the peak paraexciton density participating in the thermal component, width of the thermal component (FWHM), density of the condensate, width of the condensate (FWHM) and Heaviside step function, respectively. We assumed that the density distribution of the condensate is quadratic (Supplementary Ref. 7).

We can see in Fig. 4e that a dense signal appears around the centre of the cloud when T_mix=100 mK. This signal corresponds to the condensate, as discussed in the main text. In contrast, the distribution of the paraexciton density at other positions deviates little from that measured at T_mix=450 mK. Therefore, we

extracted the density distribution of the locally condensed signal by subtracting the distribution of the differential transmission at T_{mix} =450 mK from that at T_{mix} =100 mK using Supplementary Eqs. (42) and (45). This allowed us to estimate a paraexciton density of 4.6±0.7×10^{15} cm^{-3} (7.6±1.8×10^{15} cm^{-3}) at the bottom of the trap potential for P_{pump}=1.6 mW (8.8 mW) based on Supplementary Eq. (45).

5-d Plotting the radial profile of the density

As shown in eq. (44), we evaluated the density distribution of 1s paraexcitons within the xz plane ($n_{1s}(x, 0, z)$) from the absorption imaging. Supplementary Eq. (12) expresses that the statistical distribution of paraexcitons depends on the trap potential, which is a function of position. The trap potential, which is not isotropic, determines the position dependence of the paraexciton density. Therefore, the density distributions are slightly ellipsoidal. We extracted the radial profile of the averaged density as explained in the following to clearly visualize the emergence of an anomalous component in the statistical distribution.

First, we calculate the potential energy at a position (x_{m}, 0, z_{n}) in the trap specified by a pixel number (m, n) in the focal plane as

$$V(x_{\mathrm{m}}, y=0, z_{\mathrm{n}}) = \frac{m}{2}(\omega_x^2 x_{\mathrm{m}}^2 + \omega_z^2 z_{\mathrm{n}}^2). \tag{46}$$

Then, we discretize the potential energy with a subscript (l) as

$$V_{\mathrm{l}},\ V_{\mathrm{l}} = 0.08 \cdot \mathrm{l}^2\ \mu\mathrm{eV},\ \mathrm{l} = 0,1,2,\ldots$$

$$V_{\mathrm{l}} \leq V(x_{\mathrm{m}}, y=0, z_{\mathrm{n}}) < V_{\mathrm{l}+1}. \tag{47}$$

We evaluate the averaged density for V_{l} using the paraexciton densities at corresponding positions ($(\mathrm{m}, \mathrm{n})_{\mathrm{l}}$):

$$\hat{n}_{1\mathrm{s}}(V_{\mathrm{l}}) = \frac{\sum_{(\mathrm{m},\mathrm{n})_{\mathrm{l}}} n_{1\mathrm{s}}(x_{\mathrm{m}}, y=0, z_{\mathrm{n}})}{\sum_{(\mathrm{m},\mathrm{n})_{\mathrm{l}}} 1}. \tag{48}$$

Then, we obtain the radial profile of the averaged density as

$$V_{\mathrm{l}} = \frac{m}{2}\omega_x^2 R_{\mathrm{l}}^2, \tag{49}$$

$$\hat{n}_{1\mathrm{s}}(V_{\mathrm{l}}) = \hat{n}_{1\mathrm{s}}\left(\frac{m}{2}\omega_x^2 R_{\mathrm{l}}^2\right) = \tilde{n}_{1\mathrm{s}}(R_{\mathrm{l}}), \tag{50}$$

where R_{l} and $\tilde{n}(R_{\mathrm{l}})$ denote the radial position and the radial profile, respectively.

We plotted the radial profile of the averaged density of paraexcitons in Figs. 4d, h, i. Figs. 4h, i show that the locally condensed signal appears at radial positions below 10 μm when T_{mix} ≤400 mK. As discussed in the main text, this suggests the formation of the paraexciton condensate.

5-e Accuracy of the density

We obtained the absolute density of 1s paraexcitons using Supplementary Eq. (44). The accuracy of the

density of paraexcitons is determined by the accuracy of the dipole moment associated with the 1s-2p transition of paraexcitons shown in Supplementary Eq. (44) and the accuracy of estimating the spatial width of the entire trapped paraexitons. The combined accuracy of the density of paraexcitons is typically 20% of the density of paraexcitons. As for the former, in our prior study (Supplementary Ref. 5), we determined the dipole moment to be 3.5 ± 0.3 based on absorption spectroscopy associated with 1s-np transition. A careful analysis of Lyman series absorption strengths was necessary to determine the dipole moment, taking into account that the central cell correction due to the non-parabolicity of the valence and conduction bands causes the shrinkage of the Bohr radius of the 1s exciton state. Propagation of errors from the dipole moment provides the accuracy of the exciton density of 17 %. As for the later, the accuracy of estimating the spatial width of the entire trapped paraexitons. The accuracy of estimating the spatial width of the entire trapped paraexitons is typically 15% (see the discussion in Supplementary Note 5-f). Propagation of errors from estimations of the spatial width provides the accuracy of the density of 15 %. Therefore, the accuracy of these two parameters determines the overall accuracy of the density of paraexcitons to be 20% of the density of paraexcitons.

The sources of the statistical error in mid-infrared induced absorption imaging are a shot noise in measurements of the probe light, dark current of the MCT detector and quantization noise in the 16-bit AD converter. It typically takes several hours to obtain an image with a good noise-to-signal ratio. The reduced sample stage vibration and the high mechanical stability of our mid-infrared induced absorption imaging system allow us to perform a long-term measurement with a sufficiently small noise. A long exposure time of 5 hours results in a small shot noise and a low dark current which are about 0.001% of the signal. The quantization noise depends on the number of bits of the AD converter. The quantization noise is about 0.001% of the signal. Propagation of errors from these noises provides an uncertainty of the exciton density of 3×10^{12} cm^{-3} based on Supplementary Eq. (44). The typical density of paraexcitons is 10^{14} cm^{-3}. Therefore, the statistical uncertainty of the exciton density is about 3% of the exciton density.

5-f Estimation of the density distribution along the $[01\bar{1}]$ axis

As discussed in Supplementary Note 5-b, we needed to know the density distribution along the *y*-axis to determine the paraexciton density at the bottom of the trap potential. However, we did not measure the density distribution along the $[01\bar{1}]$ crystal axis. We measured the density distribution along the $[011]$ and the $[100]$ crystal axes. We have to estimate the density distribution along the $[01\bar{1}]$ crystal axis to determine the absolute density. Estimations of the density distribution along the $[01\bar{1}]$ crystal axis require either the distribution along the $[011]$ crystal axis or the distribution along the $[100]$ crystal axis. When we estimate the density distribution along the $[01\bar{1}]$ crystal axis based on the density distribution along the $[100]$ crystal axis, we should pay attention that the shape of the trap potential is asymmetric along the $[100]$ crystal axis. Meanwhile, the trap potential is symmetric along the $[011]$ and $[01\bar{1}]$ crystal axes.

When we estimate the density distribution along the $[01\bar{1}]$ crystal axis based on the density distribution along the $[011]$ crystal axis, we have to consider that the spatial distribution of the paraexciton density may differ in the direction perpendicular to the pump laser beam, which corresponds to the $[01\bar{1}]$ or $[100]$ crystal axis, and the direction parallel to the pump laser beam, which corresponds to the $[011]$ crystal axis. In the main text and the supplementary materials, we determined the exciton density using the density distribution along the $[011]$ crystal axis. We also evaluated the exciton density using the density distribution along the $[100]$ crystal axis in the following. As a result, these two estimations match within the accuracy of the paraexciton density. Therefore, the estimation of the distribution along the $[01\bar{1}]$ crystal using the distribution along the $[011]$ crystal axis is reasonable. We conclude that we do not have to modify figures in the main text.

We examined a difference between the spatial distribution along the direction perpendicular to the propagation direction of the pump beam and along the direction parallel to it. Supplementary Fig. 12 shows the width of the density distribution measured by induced absorption imaging along the $[011]$ and $[100]$ crystal axes at various excitation powers. The width of the density distribution along the $[011]$ ($[100]$) crystal axis is 58 μm (39 μm) at P_{pump}= 10 μW. The ratio of the spatial width along the $[011]$ crystal axis to the width along the $[100]$ crystal axis ($W_{[011]}/W_{[100]}$) is 1.5. The ratio corresponds to the ratio of the trap frequency along the $[100]$ crystal axis to the trap frequency along the $[011]$ crystal axis. The trap frequency along the $[100]$ crystal axis was 12 MHz and the trap frequency along $[011]$ crystal axis was 8 MHz. Theoretically, Supplementary Eq. (12) suggests that the width of the density distribution is inversely proportional to the trap frequency. The ratio of the width is consistent with the theoretical width calculated from Supplementary Eq. (18).

At P_{pump}= 30 mW, the ratio of the spatial with ($W_{[011]}/W_{[100]}$) is 1.8. The ratio at P_{pump}= 30 mW is 1.2 times larger than that at P_{pump} = 10 μW. Therefore, as pointed out by the reviewer, the density distribution along the direction perpendicular to the propagation direction of the pump beam is spatially localized rather than the density distribution along the parallel direction at the high excitation power of 30 mW.

We can estimate the density distribution along the $[01\bar{1}]$ crystal axis based on either the density distribution along the $[100]$ crystal axis or the density distribution along the $[011]$ crystal axis. The trap frequency along the $[01\bar{1}]$ crystal axis is the same as the trap frequency along the $[011]$ crystal axis. Therefore, we estimated the width of the density distribution along the $[01\bar{1}]$ crystal axis to be the same as the width of the density distribution along the $[011]$ crystal axis. On the other hand, the trap frequency along the $[01\bar{1}]$ crystal axis is 0.66 times as large as the trap frequency along the $[100]$ crystal axis. When we estimate the density distribution along the $[01\bar{1}]$ crystal axis based on the density distribution along the $[100]$ crystal axis, the width of the density distribution along the $[01\bar{1}]$ crystal axis is estimated to be 1.5 times as large as the width of the density distribution along the $[100]$ crystal axis. Supplementary Fig. 13 shows the estimated density at various excitation powers. The red diamonds show the estimated density based on the density distribution along the $[011]$ crystal axis. The green diamonds show the estimated

density based on the density distribution along the $[100]$ crystal axis. these two estimations match within the accuracy of the paraexciton density. Therefore, the localization of the density distribution along the $[01\bar{1}]$ crystal axis would not change a discussion on Fig. 3.

5-g Validity of bimodal fitting

We measured the paraexciton density for the localized dense signal by fitting the radial profile shown in Figs. 4 with a bimodal distribution. We performed chi-squared tests for data shown in Figs. 4 to prove the validity of bimodal fittings. Fig. 4d shows the bimodal distribution fit to radial profiles of the density at P=1.6 mW, 2.9 mW, 8.8 mW and 30 mW. Fig. 4h shows the bimodal distribution fit to a radial profile of the density at $T_{\rm mix}$=64 mK. Fig. 4i shows the bimodal distribution fit to a radial profile of the density at $T_{\rm mix}$=64 mK. We used a fitting function as follows:

$$n(\mathrm{r}) = \alpha \cdot \sqrt{1-\beta r^2} \cdot H(1-\beta r^2) + \gamma \cdot \exp[-r^2/\delta^2], \quad (51)$$

where *r, n(r), H* denote the radial position, the radial profile and Heaviside step function, respectively. α, β, γ and δ are fitting parameters. Supplementary Table 1a shows the chi-squared value of fittings. The number of data points in each radial profile is 24 and the number of fitting parameters is 4. The chi-squared value at a significance level of 0.5 is 19.3 when the degree of freedom is 20. The chi-squared value at a significance level of 0.05 is 31.41 when the degree of freedom is 20. Accordingly, the chi-squared test shown in Supplementary Table 1a suggests that fittings meet the significance level of 5% and chi-squared values are not too small.

To support our analyses, we performed Gaussian fitting using the following fitting function.

$$n(r) = \gamma \cdot \exp[-r^2/\delta^2]. \quad (52)$$

Supplementary Table 1b shows the chi-squared value of fittings. The number of data points in each radial profile is 24 and the number of fitting parameters is 2. The chi-squared value at a significance level of 0.05 is 33.92 when the degree of freedom is 22. Therefore, the chi-squared test shown in Supplementary Table 1b suggests that we have to reject the Gaussian fitting to the radial profiles of the density at a significance level of 5% except for the radial profile at $P_{\rm pump}$=1.6 mW. Chi-squared tests prove that all the density distributions are the bimodal distributions.

Supplementary Note 6. Estimation of the scattering length of paraexcitons using the Gross–Pitaevskii equation

As mentioned in the main text, the condensate has a resolution-limited width (FWHM) of 8.0±1.4 μm (7.8±1.3 μm) along the x-axis (z-axis) at $P_{\rm pump}$=1.6 mW. Meanwhile, the width of the condensate becomes 19±7 μm (13±3 μm) along the x-axis (z-axis) when $P_{\rm pump}$=8.8 mW. The excitation power dependence suggests that the exciton–exciton interaction is repulsive. Here, we used the Gross–Pitaevskii equation to

estimate the scattering length of paraexcitons:

$$\mu\Psi(\mathbf{r}) = (-\frac{\hbar^2}{2m}\nabla^2 + V(\mathbf{r}) + g_c|\Psi(\mathbf{r})|^2)\Psi(\mathbf{r}), \tag{53}$$

where $\Psi(r)$ and g_c denote the order parameter and the coupling constant. The coupling constant is expressed as

$$g_c = \frac{4\pi\hbar^2 a_s}{m}, \tag{54}$$

where a_s denotes the scattering length.

The density of the condensate is

$$n(\mathbf{r}) = |\Psi(\mathbf{r})|^2. \tag{55}$$

We approximated the equation using the Thomas-Fermi approximation:

$$\Psi(\mathbf{r}) = \sqrt{\frac{\mu - V(\mathbf{r})}{g_c}} \cdot H\left(\frac{\mu - V(\mathbf{r})}{g_c}\right), \tag{56}$$

$$n(\mathbf{r}) = \frac{\mu - V(\mathbf{r})}{g_c} \cdot H\left(\frac{\mu - V(\mathbf{r})}{g_c}\right). \tag{57}$$

The trap potential is expressed as

$$V(\mathbf{r}) = \frac{m}{2}\left(\omega_x^2 x^2 + \omega_y^2 y^2 + \omega_z^2 z^2\right), \tag{58}$$

where ω_x, ω_y, and ω_z denote the trap frequency along the x-axis, y-axis and z-axis, respectively.

Therefore, the peak density of the condensate satisfies the following equation:

$$n_c = \frac{\mu}{g_c}. \tag{59}$$

We substitute Supplementary Eqs. (58) and (59) into eq. (57):

$$n(\boldsymbol{r}) = \frac{g_c n_c - \frac{m}{2}\left(\omega_x^2 x^2 + \omega_y^2 y^2 + \omega_z^2 z^2\right)}{g_c} \cdot H\left(\frac{g_c n_c - \frac{m}{2}\left(\omega_x^2 x^2 + \omega_y^2 y^2 + \omega_z^2 z^2\right)}{g_c}\right)$$

$$= n_c\left(1 - 2\frac{x^2}{W_{xc}^2} - 2\frac{y^2}{W_{yc}^2} - 2\frac{z^2}{W_{zc}^2}\right) \cdot H\left(\frac{g_c n_c - \frac{m}{2}\left(\omega_x^2 x^2 + \omega_y^2 y^2 + \omega_z^2 z^2\right)}{g_c}\right), \tag{60}$$

where W_{xc}, W_{yc} and W_{zc} denote the width of the condensate (FWHM) along the x-axis, y-axis and z-axis. Here, the width of the condensate is expressed as

$$W_{xc} = \sqrt{\frac{4g_c n_c}{m\omega_x^2}}, W_{yc} = \sqrt{\frac{4g_c n_c}{m\omega_y^2}} \; and \; W_{zc} = \sqrt{\frac{4g_c n_c}{m\omega_z^2}}. \tag{61,62 and 63}$$

We reproduced the widths of 19±7 μm (13±3 μm) along the x-axis (z-axis) using a scattering length of

4±2 nm (4±2 nm), a measured peak density of $7.6\pm1.8\times10^{15}$ cm^{-3} and trap frequencies of 8.4 MHz along the x-axis and 12.4 MHz along the z-axis.

Supplementary References

1. Yoshioka, K., Morita, Y., Fukuoka, K. & Kuwata-Gonokami, M. Generation of ultracold paraexcitons in cuprous oxide: A path toward a stable Bose-Einstein condensate. *Phys. Rev. B* **88**, 041201 (R) (2013).

2. Trauernicht, D. P., Wolfe, J. P. & Mysyrowicz, A. Thermodynamics of strain-confined paraexcitons in Cu_2O. *Phys. Rev. B* **34**, 2561-2575 (1986).

3. Kreingold, F. I. & Makarov, V. L. Investigation of the mechanism of the deformation stimulation of a paraexciton level in the luminescence of cuprous oxide crystals. *Sov. Phys. Semicond.* **8**, 962-965 (1975).

4. Yoshioka, K., Morita, Y., Fukuoka, K. & Kuwata-Gonokami, M. Generation of ultracold paraexcitons in cuprous oxide: A path toward a stable Bose-Einstein condensate. *Phys. Rev. B* **88**, 041201 (R) (2013).

5. Yoshioka, K., Ideguchi, T., Mysyrowicz, A. & Kuwata-Gonokami, M. Quantum inelastic collisions between paraexcitons in Cu_2O. *Phys. Rev. B* **82**, 041201(R) (2010).

6. Morita, Y., Suzuki, H., Yoshioka, K. & Kuwata-Gonokami, M. Observation of ultrahigh mobility excitons in a strain field by space-and time-resolved spectroscopy at subkelvin temperatures. *Phys. Rev. B* **100**, 035206 (2019).

7. Pitaevskii, L. & Sringari, S. Ed., *Bose-Einstein condensation and superfluidity* (Oxford University Press, 2016).
